# β-Ginkgotides: Hyperdisulfide-constrained peptides from *Ginkgo biloba*

**DOI:** 10.1038/s41598-017-06598-x

**Published:** 2017-07-21

**Authors:** Ka H. Wong, Wei Liang Tan, Tianshu Xiao, James P. Tam

**Affiliations:** School of Biological Sciences, Nanyang Technological University, Singapore, 637551 Singapore

## Abstract

Hyperdisulfide-constrained peptides are distinguished by their high stability and diverse functions. Thus far, these peptides have been reported from animals only but their occurrence in plants are rare. Here, we report the discovery, synthesis and characterization of a hyperdisulfide-constrained peptides family of approximately 2 kDa, β-ginkgotides (β-gB1 and β-gB2) from *Ginkgo biloba*. Proteomic analysis showed β-ginkgotides contain 18‒20 amino acids, of which 16 residues form a conserved six-cysteine core with a highly clustered cysteine spacing of C‒CC‒C‒CC, an arrangement that has not been reported in cysteine-rich peptides. Disulfide mapping revealed a novel disulfide connectivity of CysI‒IV, CysII‒VI and CysIII‒V. Oxidative folding of synthetic β-gB1 to the native form was obtained in 70% yield. The synthetic β-gB1 displays a compact structure with no regular secondary structural elements, as determined by NMR spectroscopy. Transcriptomic analysis showed precursor *βgb1* has a four-domain architecture and revealed an additional 76 β-ginkgotide-like peptides in 59 different gymnosperms, but none in angiosperms. Phylogenetic clustering analysis demonstrated β-ginkgotides belong to a new cysteine-rich peptide family. β-Ginkgotide is resistant to thermal, chemical and proteolytic degradation. Together, β-ginkgotides represent the first-in-class hyperdisulfide-constrained peptide family from plants with a novel scaffold that could be useful for engineering metabolically stable peptidyl therapeutics.

## Introduction

Plants have evolved complex and effective defense mechanisms that release pathogenesis-related biomolecules against pathogen attacks^1^. These chemicals range from small-molecule metabolites to large biomolecules and are natural products that have been essential for drug discovery. Among the pathogenesis-related biomolecules, plant cysteine-rich peptides (CRPs), which fall in the chemical space of 2–6 kDa, are putative active compounds in medicinal herbs. A common characteristic of such plant CRPs is their stable structures, which often contain three to five intramolecular disulfides as cross-braces to render them exceptionally resistant to thermal and proteolytic degradation. In addition, the CRPs in this chemical space are promising therapeutic candidates because they possess a large footprint that could increase their on-target specificity and minimize their off-target side reactions^2^. These exceptional features, coupled with their underrepresentation as active compounds in herbal medicine, have prompted our laboratory to develop an herbalomics program to discover the CRPs in medicinal herbs that can be potential drug candidates^1, 3–6^.

*Ginkgo biloba*, commonly known as the maidenhair tree or YinXing in Chinese, is a deciduous gymnosperm native to Southwestern China. The *G*. *biloba* nut is used as a traditional Chinese medicine for relieving symptoms of asthma, cough, vaginal discharge and urination discomfort^7^. Apart from their medicinal use, *G*. *biloba* nuts are commonly used as a functional food in Asian cuisines. Major phytochemicals in *G*. *biloba* nuts include alkyl phenols, cyanophoric glycosides, flavonoids and terpene lactones^8, 9^. Lipid extracts of *G*. *biloba* nuts were shown to reduce apolipoprotein B secretion and enhance low-density lipoprotein receptor expression in human hepatoma cells^10^. Furthermore, oral supplementation of *G*. *biloba* nuts for four weeks has been shown to reduce hepatic cholesterol and triacylglyceride level in mice^10^. Nonetheless, limited information is available regarding the occurrence and therapeutic potential of CRPs in *G*. *biloba* nuts, an area that is of interest to our laboratory. Recently, we have characterized eleven proline-rich 8C-hevein-like peptides, ginkgotides from *G*. *biloba* leaves. The leaf-derived ginkgotides display potent anti-fungal activity against common phyto-pathogenic strains^5^.

**Figure 1:**
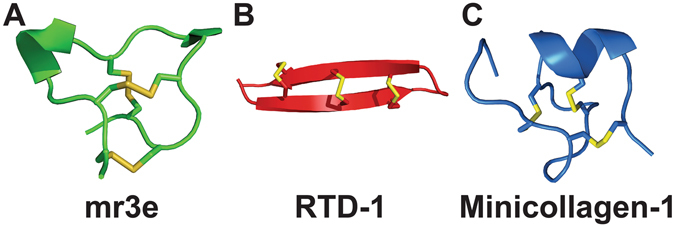
Animal hyperdisulfide-constrained peptides. (**A**) M-1 conotoxin mr3e from the marine cone snail *Conus marmoreus* (PDB: 2EFZ), (**B**) θ-defensins RTD-1 from *Rhesus macaque* (PDB: 2LYF) and (**C**) minicollagen-1 from the jellyfish *Hydra sp*. (PDB: 1SOP).

CRPs are grouped into families based on their cysteine motifs and disulfide connectivities. Based on their disulfide connectivities, the CRPs can be broadly classified into two types: cystine knots which are exemplified by knottins, and symmetrics which are represented by thionins^1^. Recently, our laboratory discovered two new families of CRPs with novel disulfide connectivity, the jasmintides^11^ and the lybatides. The jasmintides, which contain 27 amino acids with a molecular weight of 3.1 kDa, are six-cysteine CRPs (6C-CRPs) from *Jasminum sambac* with a disulfide connectivity of CysI‒V, CysII‒IV and CysIII‒VI^11^. Lybatides, which are 33 amino acids in length, are eight-cysteine CRPs from the *Lycium barbarum* root cortex with a disulfide connectivity of CysI‒VI, CysII‒VIII, CysIII‒VII and CysIV‒V. Thus far, the knottins, with a disulfide connectivity of CysI‒IV, CysII‒V and CysIII‒VI, are the most frequently encountered 6C-CRPs in plants. In addition, the structure of CRPs becoming increasingly constrained with decreasing molecular weight. CRPs become hyperdisulfide-constrained when their cysteine residues account for >30% of total amino acids within the cystine core. Thus far, hyper-disulfide-constrained CRPs had been found in animal kingdom and their occurrence in planta have not been reported. Figure 1 exemplifies the animal hyperdisulfide-constrained peptides, including M-1 conotoxin mr3e from the marine cone snail *Conus marmoreus* ^12^, θ-defensins RTD-1 from *Rhesus macaque* ^13^ and minicollagen-1 from the jellyfish *Hydra* sp.^14^.

Herein, we report the discovery, characterization and synthesis of a new family of hyperdisulfide-constrained peptides ginkgotides, β-ginkgotides β-gB1 and β-gB2, from the nuts of *G*. *biloba*. The nut-derived ginkgotides and leaf-derived, proline-rich 8C-hevein-like peptide family of ginkgotides are renamed the α-ginkgotides based on the order of their discovery^5^. The β-ginkgotides differ from the α-ginkgotides in length, sequence composition, cysteine spacing and disulfide connectivity. Strikingly, the β-ginkgotides are small, 18‒20 amino acids in length, and hyperdisulfide-constrained, with six cysteine residues arranged in a novel CC‒C‒CC‒C motif. A structure determination by NMR showed that β-gB1 displays a novel disulfide connectivity and a compact structure with no helical or beta strand secondary structural elements. Transcriptomic data mining found an additional 76 β-ginkgotide-like peptides in gymnosperms but not in angiosperms. A neighbor-joining clustering analysis revealed that β-ginkgotides represent a new family of CRPs that are only distributed in gymnosperms. Taken together, the discovery of β-ginkgotides expands the occurrence and distribution of CRPs *in planta* and provides insight into the molecular diversity of CRPs in modern gymnosperms and angiosperms.

## Results and Discussion

### β-Ginkgotide: Identification and Purification

**Figure 2:**
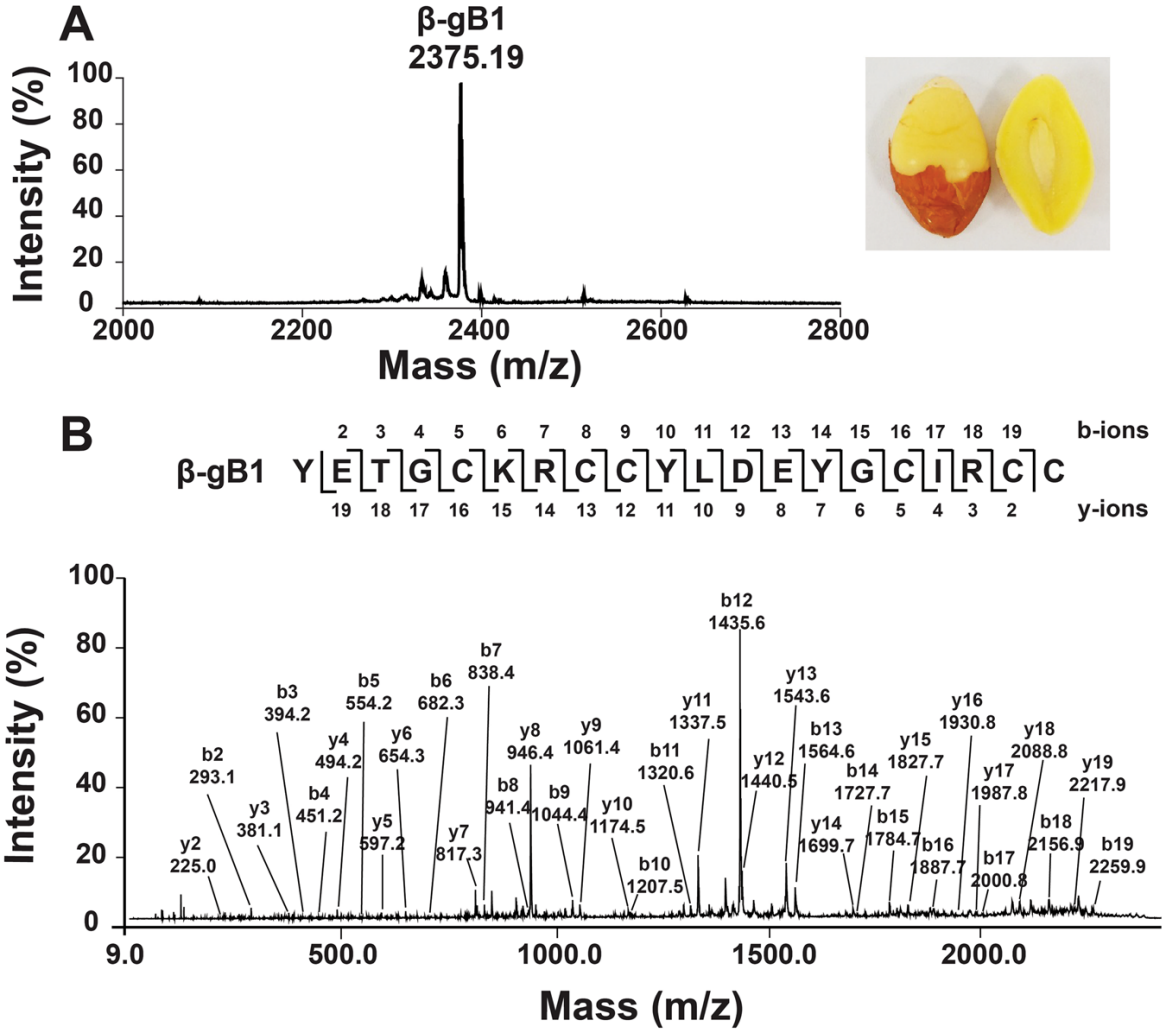
Mass spectra of *Ginkgo biloba* nut. (**A**) MALDI-TOF MS profile of the *G*. *biloba* nuts in a mass range of 2000−2800 Da. *G*. *biloba* nuts (5 g) were extracted with boiling water (50 mL) and fractionated by solid-phase extraction C_18_ column. (**B**) MALDI-TOF/TOF MS/MS profile of the *G*. *biloba* nuts in a mass range of 10–2500 Da. The purified β-ginkgotide β-gB1 was reduced by 20 mM dithiothreitol and alkylated with 200 mM iodoacetamide. Assignment of isobaric amino acids such as Leu/Ile were confirmed by the transcriptome data.

A mass-spectrometry-driven approach was employed to identify putative CRPs that are present in medicinal plants. *G*. *biloba* nuts (5 g) were extracted with boiling water (50 mL), and the extraction was fractionated by solid-phase extraction using a C_18_ column and profiled by MALDI-TOF MS in the mass range between 2 and 6 kDa. Figure 2A shows a mass spectrum with peaks between 2.0 and 2.8 kDa. A compound with a molecular mass [M + H]^+^ of 2375.19 Da was isolated and designated β-ginkgotide β-gB1. In a scaled up purification using 2 kg of *G*. *biloba* nuts, β-gB1 and an additional β-ginkgotide β-gB2 with a molecular mass [M + H]^+^ of 2083.04 Da were isolated using a series of chromatography steps. The extraction yields of β-gB1 and β-gB2 were approximately 3 and 0.1 mg, respectively, per kg of dried *G*. *biloba* nuts.

To determine the number of cysteine residues that are present in the β-ginkgotides, a mass shift experiment was performed. β-Ginkgotide β-gB1 and β-gB2 were reduced by dithiothreitol and subsequently alkylated by iodoacetamide. The disulfide content of β-ginkgotide was determined by comparing the mass difference before and after the S-alkylation using MALDI-TOF MS. The β-ginkgotide displayed a mass shift of 348 Da, and each S-alkylated Cys causes a mass increase of 58 Da, suggesting that these peptides contained six Cys residues (Figure S1, Supporting information).

### Primary Sequence Determination

*De novo* sequencing was performed to determine the primary sequence of the S-alkylated β-gB1 using MALDI-TOF/TOF MS/MS. The amino acid assignment was based on the *b*- and *y*-ions detected during the tandem MS fragmentation (Fig. 2B). The MS/MS profile of β-gB1 revealed a putative sequence of YETGC*X*RCCY*X*DEYGC*X*RCC, where *X* represents the isobaric residues Ile/Leu or Lys/Gln. Their assignment was confirmed with the cDNA sequence from OneKP (accession number: SGTW-2035618) to give the complete sequence of β-gB1 as YETGCKRCCYLDEYGCIRCC. Furthermore, the predicted molecular mass (2373.91 Da) was in agreement with the experimental molecular mass of β-gB1 (2374.19 Da) that was determined from the MS spectrum. The amino acid sequence of β-gB2 was determined in the same manner. β-Ginkgotide β-gB2 is a truncated β-gB1 with two amino acids (Tyr1 and Glu2) omitted at the N-terminus. The β-ginkgotide β-gB1 was chosen as the representative for further characterization because of its high abundance.

### Disulfide Connectivity

**Figure 3:**
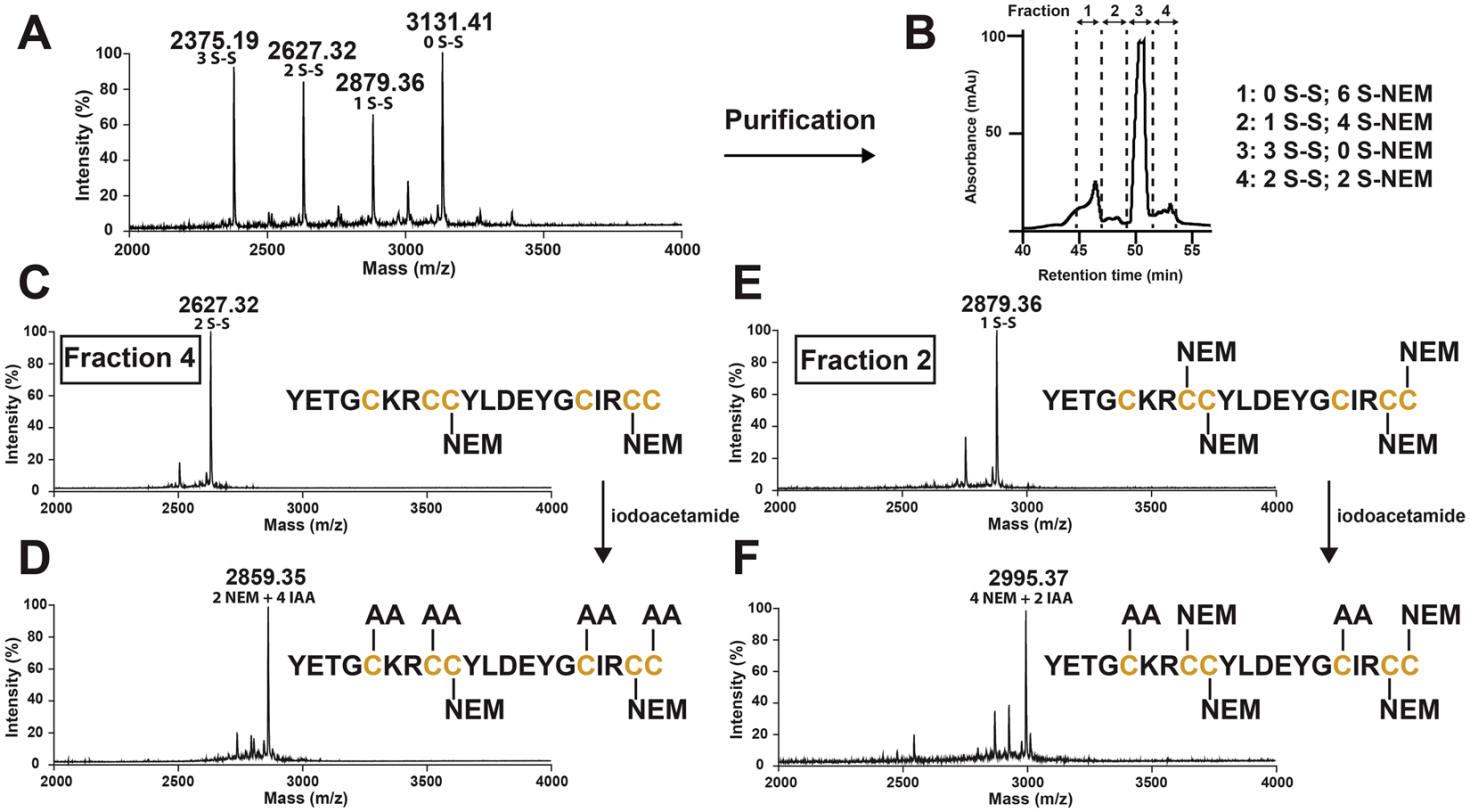
Disulfide mapping of β-ginkgotide β-gB1. (**A**) MALDI-TOF MS profile of partially alkylated β-gB1. The purified β-gB1 was partly S-reduced by tris(2-carboxyethyl)phosphine for 17 min and S-alkylated by N-ethylmaleimide (NEM) for 1 hr. (**B**) Chromatogram of partially alkylated β-gB1. The reaction mixture was separated by HPLC and four major fractions were collected. These fractions were profiled by MALDI-TOF MS to determine the number of disulfide bonds present. (**C**) MALDI-TOF MS profile of fraction 4 with two-N-ethylmaleimide alkylated intermediate and (**D**) fully reduced species. (**E**) MALDI-TOF MS profile of fraction 2 with four-N-ethylmaleimide alkylated intermediate and (**F**) fully reduced species. The two- and four-N-ethylmaleimide alkylated intermediate species were further reduced with dithiothreitol and alkylated with iodoacetamide. The fully alkylated β-ginkgotide was desalted and sequenced by MALDI-TOF/TOF MS/MS.

The disulfide connectivity was determined by partially reducing β-gB1 with tris(2-carboxyethyl)phosphine to generate intermediates with one or two disulfide bonds remaining intact. These intermediates were immediately alkylated by N-ethylmaleimide (NEM) under acidic conditions (pH 3.5) to prevent disulfide bond scrambling and were subsequently purified by RP-HPLC. The number of NEM-labeled cysteine (S-NEM) residues were determined by MALDI-TOF MS (Fig. 3A). Each NEM-labeled cysteine causes a mass increase of 126.15 Da; intermediates that displayed shifts of 252.30 and 504.60 Da thus had one and two reduced disulfide bonds, respectively. Figure 3B shows four fractions that were collected from the HPLC, including fraction 1, which had six S-NEM and no intact disulfide bonds (0S-S); fraction 2, which had four S-NEM and one intact disulfide bond (1S-S); fraction 3, which had native β-gB1 with three intact disulfide bonds (3S-S); and fraction 4, which had two S-NEM and two disulfide bonds (2S-S). Fractions 2 and 4 were then fully reduced by dithiothreitol and alkylated with iodoacetamide. The positions of the NEM- and acetamido-labeled cysteines (S-AA) were determined by tandem MS fragmentation and employed to deduce the peptide’s disulfide connectivity. Figure 3C–F summarize our disulfide mapping results. The 2S-S intermediate revealed the CysIII‒V disulfide linkage, and the 1S-S intermediate revealed both CysII‒VI and CysIII‒V. The third disulfide linkage, from CysI‒IV, was obtained by deduction.

Currently, the disulfide connectivity of plant CRPs with six cysteine residues can be arbitrarily classified topologically into three families. They are (1) cystine knot peptides (CysI‒IV, CysII‒V and CysIII‒VI)^15^, (2) symmetrics (CysI‒VI, CysII‒V and CysIII‒IV)^16^ and (3) jasmintides (CysI‒V, CysII‒IV and CysIII‒VI)^11^. Cystine knot peptides, including cystine knot α-amylase inhibitors (CKAIs), 6C-hevein-like peptides, carboxypeptidase inhibitors and cyclotides, represent the majority of known disulfide connectivities of plant CRPs by far^17^. These peptides share a similar topology of three disulfide bonds; one disulfide bond penetrates through a macrocycle formed by the other two disulfide bonds, which interlock the peptide backbone to form a knotted structure^15^. The symmetric family of disulfide connectivities is found in thionin and thionin-like peptides, which also contain a symmetric cysteine motif. The symmetry disulfide pattern in thionins consists of the first cysteine connecting to the last one, the second cysteine to the second to last one, and so on^1^. The third family, the jasmintides, is a variation of the symmetrics, but the presence of consecutive cysteine residues in a CC motif at their C-terminus alters the symmetric disulfide connectivity. In the jasmintides, the disulfide bonds CysII‒IV and CysIII‒VI cross-brace with each other at the C-terminus, while the disulfide bond CysI‒V brings the C-terminus and N-terminus into close proximity^11^. Finally, the disulfide connectivity of CysI‒IV, CysII‒VI and CysIII‒V that is found in β-gB1 represents the fourth family of disulfide connectivities that has not been reported in any plant CRP families.

The reductive unfolding order reflects the exposure of the disulfide bonds to the solvent and reducing reagents. The solvent-exposed outer disulfide bonds are more labile to reducing agents than those buried inside the cystine core. Interestingly, the unfolding order in β-gB1 was CysIII‒V and CysII‒VI, followed by CysI‒IV, which is different from that of allotide Ac2, a CKAI from *Allamanda cathartica* leaves, and cliotide T2^4^, a cyclotide from *Clitoria ternatea* flowers and leaves; in these cases, the most buried disulfide bond was the CysII‒V and the CysIII‒VI, respectively^3^. The solvent-exposed outer disulfide bonds in β-gB1 (CysIII‒V and CysII‒VI) are labile to reducing agents compared with the CysI‒IV bond, which is buried inside the core.

### Oxidative Folding of β-gB1

To determine the oxidative folding conditions, the reduced form of synthetic β-gB1 was obtained by solid-phase peptide synthesis and purified by reversed-phase HPLC. Synthetic β-gB1 was subjected to oxidative folding in 13 conditions with different concentrations of co-solvent, redox reagents and duration, as shown in Table S1, Supporting information. All experiments were carried out in ammonium bicarbonate buffer at a pH of 8.5^18^. For the redox reagents, the use of the standard redox pair of reduced and oxidized glutathione (4:400 mM) produced a higher yield of 38.8% (Run 2) than the use of the less expensive redox pair of cysteineamine and cystamine (4:400 mM), which gave a yield of 28.9% (Run 1). Adding dimethyl sulfoxide (10% v/v) to the folding solution increased the oxidative folding yield from 38.8 (Run 3) to 55.3% (Run 4). This result agreed with our previous experience in the oxidative folding of cyclotides in which dimethyl sulfoxide served as a mild oxidizer and a strong aprotic polar solvent that minimized peptide aggregation^18, 19^. However, the use of isopropanol (20% v/v) as an organic co-solvent to enhance the conformational stability to aid folding was unsuccessful. The folding yield was reduced to 17.4% (Runs 5 and 6), suggesting that the addition of organic solvent is unnecessary. To evaluate the effects of the ratios of redox reagents on the folding yield, three different concentrations of reduced and oxidized glutathione (4:400, 4:800 and 2:400 mM) were used. An increase of the reduced-to-oxidized glutathione ratio increased the folding yield (Runs 9 and 13), while the highly reducing conditions that were present at a ratio of 2:400 mM produced the highest yield, 73.4% (Run 13). The oxidative folding apparently became efficient once the correct mix of redox reagents were determined, as the incubation time (2, 6 and 24 hr) had little effect; prolonging the folding reaction beyond 2 hr did not show significant improvement in yield. Together, the optimized folding conditions for β-gB1 are as follows: oxidative folding was initiated at room temperature by adding 0.1 mg of synthetic, reduced β-gB1 to 1 mL of ammonium bicarbonate buffer (0.1 M at pH 8.5) that contained 2 mM reduced glutathione, 400 μM oxidized glutathione and 10% (v/v) dimethyl sulfoxide, which was then incubated for 2 hr. The folding yield of the synthetic β-gB1 under such optimized conditions was 76.7%. The synthetic β-gB1 co-eluted with the native β-gB1 (Figure S2, Supporting information), suggesting that they folded similarly. In addition, NMR structural characterization was used to confirm the disulfide connectivity of synthetic β-gB1.

### NMR Structure

**Figure 4:**
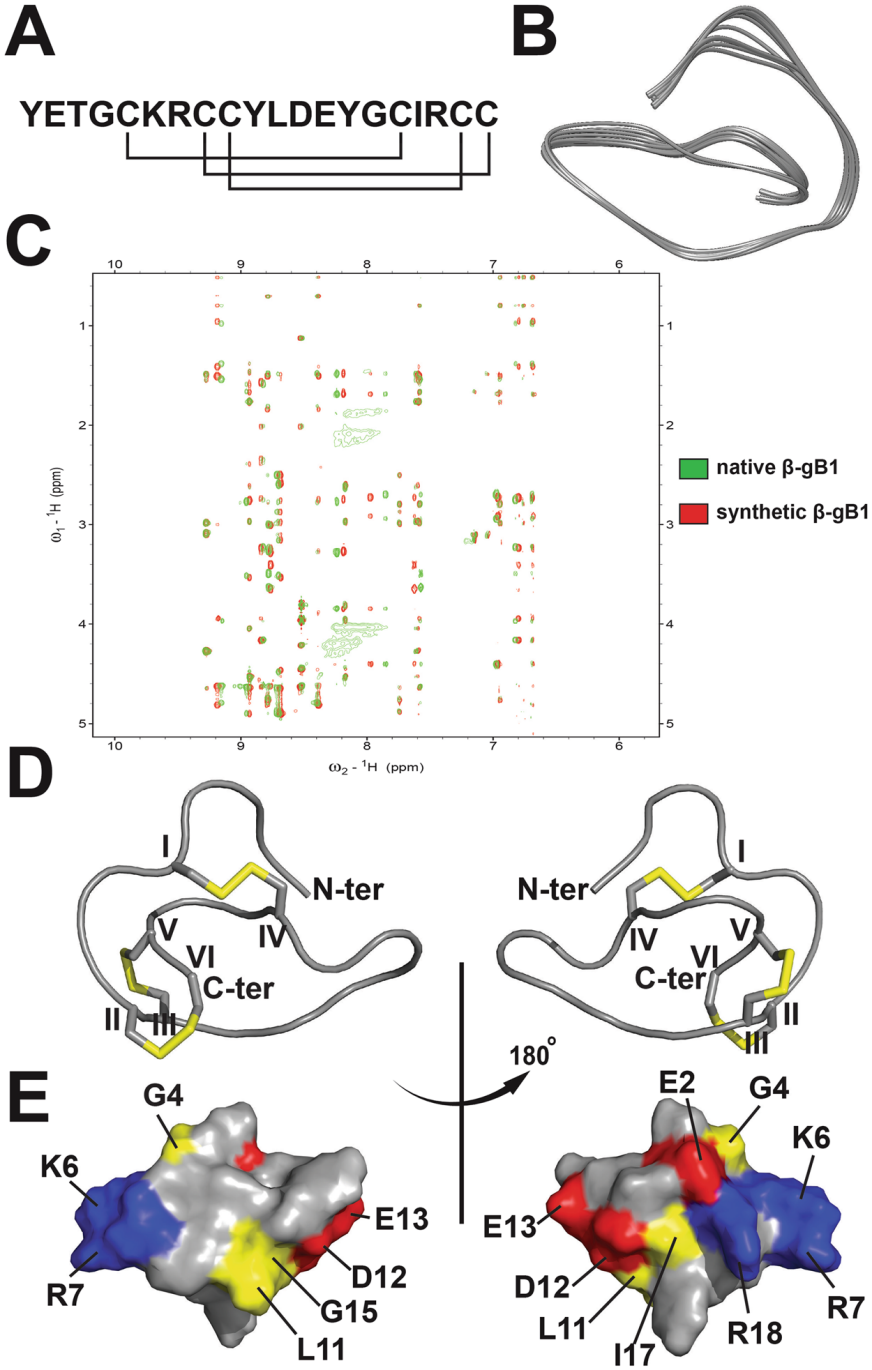
NMR spectra and structures of β-gB1 (PDB: 5XIV). (**A**) Sequence and disulfide connectivity of β-gB1. (**B**) The 20 lowest energies ensembles of β-gB1. (**C**) Overlapped 2D NOESY spectra of native (green) and synthetic β-gB1 (red) displayed by Sparky 3.115. (**D**) The ribbon representation of the synthetic β-gB1 structure. The disulfide bonds are formed between CysI–CysIV, CysII–CysVI, CysIII–CysV. (**E**) Surface topology of the synthetic bL1 structure. The distribution of electrostatic charges was illustrated in red (negatively charged), blue (positively charged) and yellow (hydrophobic). The positively charged surface (K6, R7 and R18) was opposed to the negatively charged surface (E2, D12 and E13).

To evaluate whether the synthetic β-gB1 has the same peptide fold as the native protein, their 2D NOESY spectra were measured. As shown in Fig. 4A, most of the NOE cross peaks from both the synthetic and native forms (highlighted in green and red, respectively) overlapped, suggesting that the synthetic β-gB1 shared the same peptide fold as the native β-ginkgotide. Subsequently, the synthetic β-gB1 was employed for NMR characterization, which was performed using a combination of 2D ^1^H-^1^H-TOCSY and ^1^H-^1^H-NOESY (Tables S2 and S3, Supporting information). An unambiguous assignment was achieved based on the NOE cross peaks between the amide protons (HNi) and the side chain protons from the previous residue (Hα_i-1_, Hβ_i-1_ and HΥ_i-1_ if any), as well as the patterns of the side chain peaks in the TOCSY spectrum. The NMR structure of β-gB1 was determined from a total of 206 NMR-derived distance restraints and 9 dihedral angle restraints. The ensemble of the 20 lowest-energy structures had a root-mean-square deviation of 0.74 ± 0.30 Å and 1.77 ± 0.44 Å for the backbone and heavy atoms, respectively. Figure 4B shows the backbone traces of 20 ensembles with the lowest energies. The spin-spin systems of β-gB1 were identified and all proton resonances were unambiguously assigned. Figure S3 (Supporting information) shows the complete amide proton assignment of the NOESY spectrum. A PROCHECK analysis indicated that all residues were distributed in the allowed region of the Ramachandran map. Overlapped NOESY spectra of the native and synthetic β-gB1 showed five slightly shifted amide proton peaks (Fig. 4C), corresponding to residues D12, E13, Y14, G15 and I17, all of which are located in close proximity and in an acidic face of the molecule (Fig. 4E). The peak shift could be due to the interaction between these residues and impurities, most likely cationic secondary metabolites derived from the plant sample during our purification process. As shown in Fig. 4D, the structure of synthetic β-gB1 consisted of three turns with no defined secondary structure.

To determine the disulfide connectivity of the synthetic β-gB1, the averaged overall energies of 15 different disulfide bond combinations were assessed by CNSsolve 1.3 and are summarized in Table S4, Supporting information. Figure S4 (Supporting information) shows the NOESY spectrum with NOEs that confirm the assigned pattern of disulfide bonds The different disulfide bond patterns were assumed for structure calculations in which the same distance and dihedral angle restraints were uploaded for simulated annealing. In each combination, 20 structures with the lowest overall energies were selected. The energies of the structures of the 15 disulfide combinations illustrated that pattern 1 had a lower energy (357.55 ± 5.56 kcal/mol) than all the other disulfide bond patterns, which displayed energies ranging from 466.59 ± 10.69 to 1004.41 ± 62.54 kcal/mol (Table S4, Supporting information). In comparison, the structural energies for the disulfide connectivity of the cystine knot peptide (pattern 2), thionin (pattern 15) and jasmintide (pattern 9) were 727.12 ± 178.71, 466.59 ± 10.69 and 471.87 ± 17.20 kcal/mol, respectively, suggesting that pattern 1 is the most stable disulfide arrangement and significantly different from other reported CRP families. As illustrated in Fig. 4D, the disulfide bond between Cys5 and Cys16 connects the N-terminus to the-C terminus. The disulfide bonds Cys8‒Cys20 and Cys9‒Cys19 bring the C-terminus into proximity with the cystine core. These results suggested that β-gB1 possesses a novel disulfide connectivity of CysI‒IV, CysII‒VI, and CysIII‒V, which agrees with the disulfide connectivity that was determined by chemical means using the aforementioned partial alkylation. The three-dimensional structure revealed that β-gB1 is bipolar and dumbbell-shaped, with a positive and a negative patch located at opposite ends, connected by a hydrophobic bar. We speculate that this arrangement may facilitate the interaction of β-ginkgotides with charged glycoproteins. This hypothesis was supported by our preliminary experiments, which shows that β-ginkgotide binds to heparin beads (data not shown). In addition, our recent studies suggest that such hyperdisulfide-constrained peptides are cell permeable and could interact with intercellular proteins^20, 21^.

Figure 4E shows the surface topology of β-gB1; positive, negative and hydrophobic residues were highlighted in blue, red and yellow, respectively. The positively charged residues (Lys6, Arg7 and Arg18) were located on the opposite surface of the negatively charged (Glu2, Asp12 and Glu13) residues, while the hydrophobic residues are concentrated in the core of the molecule. The pairwise structural alignment algorithm TM-align was used to determine whether β-gB1 folded similarly to members of the other CRP families, and its results were summarized in Table S5, Supporting information. A TM-score <0.3 means that the two candidates have random structural similarity, while a value >0.5 means that they shared the same protein fold. β-Ginkgotide β-gB1 has an average TM-score value of 0.14, 0.14, 0.15, 0.16 and 0.18 compared to the thionins, carboxypeptidase inhibitors, CKAIs, 6C-hevein-like peptides and jasmintides, respectively. Together, these results suggested that β-gB1 has a novel protein fold that is significantly different from those of the other reported CRP families, mostly due to the presence of an unusual cysteine spacing and the unique disulfide connectivity.

### Comparison of Cysteine Frameworks in 6C-CRP Families

**Figure 5:**
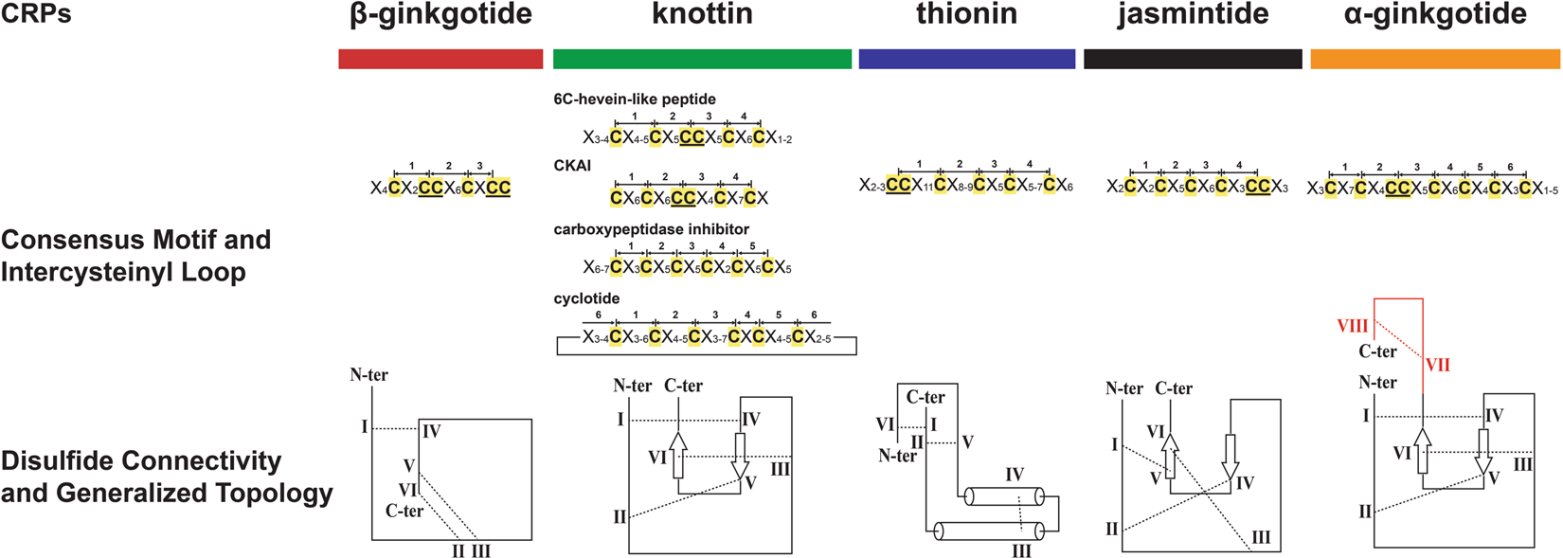
Cysteine framework comparison of plant CRPs with six cysteine residues. β-Ginkgotide β-gB1 displayed a novel cysteine spacing of C–CC–C–CC, with the presence of two CC motif forming three intercysteinyl loops as compared to other CRPs consisting four to six intercystinyl loops. β-Ginkgotide β-gB1displayed a novel disulfide connectivity of CysI–CysIV, CysII–CysVI, CysIII–CysV, which differed from other known CRPs such as cystine knot peptides (CysI‒CysIV, CysII‒CysV and CysIII‒CysVI), thionins (CysI‒CysVI, CysII‒CysV and CysIV‒CysVI) and jasmintides (CysI‒CysV, CysII‒CysIV and CysIII‒CysVI). Intercysteinyl cysteine content refers to the percentage of cysteine present within the cystine core (between the first and last cysteine), in which β-ginkgotide β-gB1displayed the highest content among others.

The term “cysteine framework” refers to the cysteine spacing and disulfide connectivity in CRPs. Cysteine spacing defines the positions of the cysteines within a peptide and the number of amino acids between cysteine residues, whereas the disulfide connectivity defines how the cysteine residues connect in a three-dimensional space. Figure 5 compares different schematic disulfide-constrained frameworks of the β-ginkgotides and the 6C-CRPs, CRPs with six cysteine residues.

The cysteine framework of β-ginkgotides impacts their structure in two ways. First, it alters their loop size. The amino acids between successive cysteine residues divide the peptide backbone into segments, commonly referred as loops. Cystine knot peptides comprised the majority of CRPs have a cysteine spacing of either C‒C‒CC‒C‒C, found in 6C-hevein-like peptides and CKAIs as 4-loop CRPs, or C‒C‒C‒C‒C‒C, found in carboxypeptidase inhibitors as 5-loop CRPs. Cyclotides which are head-to-tail cyclized knottins with a C‒C‒C‒C‒C‒C cysteine spacing are 6-loop CRPs. Compared to these four to six looped cystine-knot peptides, β-ginkgotides, with a cysteine spacing of C‒CC‒C‒CC, only have three loops because of the presence of two CC motifs (two adjacent cysteine residues). The presence of a CC motif can be found in various CRPs such as CKAIs, thionins and jasmintides; however, the novel cysteine spacing in β-gB1 has not yet been observed in other known plant CRPs. For example, the CC motif in 6C-hevein-like peptides and CKAIs is located at CysIII and CysIV, whereas in thionins, the CC motif is located near the N-terminus, at CysI and CysII. Interestingly, the cysteine spacing of jasmintides is a mirror image of that of thionins, whose CC motif is located near the C-terminus, at CysV and CysVI. Since each family CRPs share a common structural fold, the size and sequence variations of the displaying loops play an important role in diversifying their biological functions.

Second, the cysteine framework in β-ginkgotides impacts their cystine-core constraint. β-Ginkgotides have a relatively small intercysteinyl distance, with 16 amino acid residues between the first and sixth cysteine residue and an average cysteine content of 37.5% (number of cysteines per cysteine core). That translates to more than one out of every three residues that are located in the cysteine core (starting from the first to the last cysteine) being a cysteine. In comparison, the number of intercysteinyl residues in carboxypeptidase inhibitors, 6C-hevein-like peptides, CKAIs, cyclotides, thionins and jasmintides are 27, 30, 25, 22–26, 35–38 and 22 residues, with an average cysteine content of 22.2, 24.0, 20.7, 25.2, 16.5 and 27.3%, respectively. Thus, the 37.5% average cysteine content in β-ginkgotides, compared with the known range of 16.5–27.3% of the other 6C-CRP families, suggests that the β-ginkgotides are hyperdisulfide-constrained.

To determine the occurrence of the β-ginkgotide disulfide connectivity among different kingdoms, a public database search was performed, including searches of the Protein Data Bank^22^, Cybase^23^, PhytAMP^24^, YADAMP^25^, Knottin Database^26^, ATDB^27^, ConoServer^28^ and the ArachnoServer^29^. A total of 65 6C-CRPs in the mass range of 2.0‒3.0 kDa with a confirmed disulfide framework were found. Conotoxins with a disulfide spacing of CC‒C‒C‒CC and a cystine knot connectivity were the most common framework, followed by the cyclotide disulfide spacing of C‒C‒C‒C‒C‒C, with a cystine knot, and the θ-defensin disulfide spacing of CC‒C‒C‒CC, with a symmetric disulfide connectivity. However, no cysteine framework similar to that of β-ginkgotide was found in these databases, suggesting that the β-ginkgotide disulfide connectivity is novel not only among CRPs in plants but also among those in animals.

### Transcriptomic Data Mining of β-Ginkgotide Homologs *In Planta*

The full-length β-ginkgotide precursor sequence, namely, *β*-*gb1*, which encoded β-gB1 and β-gB2, was obtained from the OneKP transcriptome database^30^. A tBALSTn using the β-gB1 sequence as a query was performed to determine the distribution of its homologs *in planta*. The search results revealed 101 sequence homologs from 67 different plants. A manual filtering was employed to remove the precursor sequences that consisted of incomplete sequences with a signal peptide <10 amino acids, identical sequences from the same plant and sequences with an odd number of cysteine residues. Subsequently, a total of 76 putative β-ginkgotide homologs were identified in 59 plants (Table S6, Supporting information). Ten of the putative mature peptides were expressed in multiple plants, which were composed of identical mature peptides that had different propeptides and/or C-terminal tails. For example, aC1 was expressed in ten different plants from the same plant family (Cupressaceae), including *Austrocedrus chilensis*, *Calocedrus decurrens*, *Fokienia hodginsii*, *Juniperus scopulorum*, *Microbiota decussata*, *Neocallitropsis pancheri*, *Papuacedrus papuana*, *Pilgerodendron uviferum*, *Callitris gracilis* and *Callitris macleayana* (Table S6, Supporting information). This finding is consistent with the occurrences of the natural products, that is, the same product can be found in different species or families.

**Figure 6:**
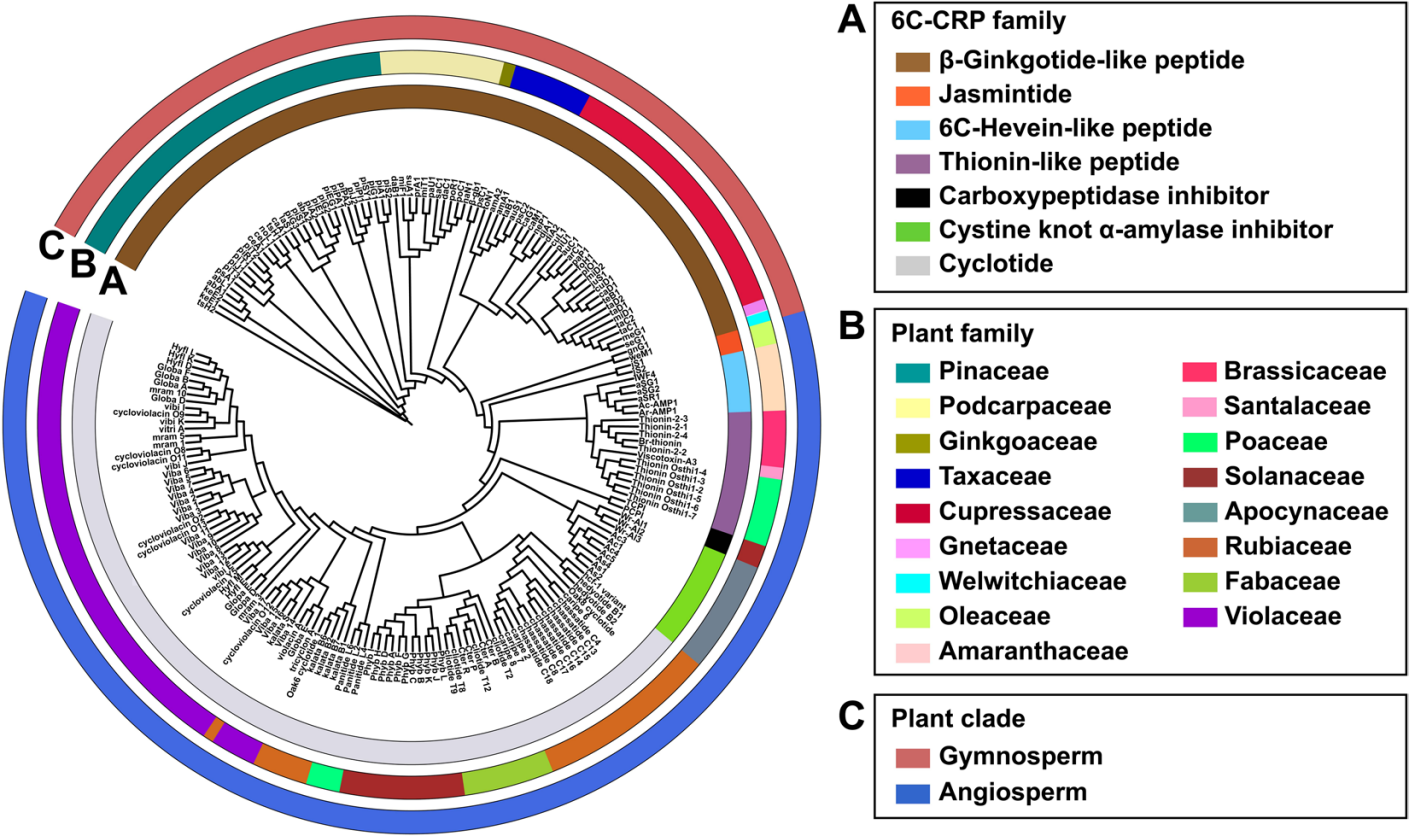
Phylogenetic tree of β-ginkgotide β-gB1, β-ginkgotide-like peptides and other reported CRPs with six cysteine residues. The precursor sequences β-gB1, β-ginkgotide-like peptides, jasmintides, 6C-hevein-like peptides, thionins, carboxypeptidase inhibitors, cystine knot α-amylase inhibitors and cyclotides were aligned by Kalign and the phylogenetic tree was generated by iTOL. Classification based on (**A**) the number of cysteine residues in the mature domain, (**B**) plant’s family and (**C**) plant’s clade.

To determine the similarity between the β-ginkgotide-like peptides and the other CRPs, their full precursor sequences were aligned using Kalign and analyzed using a neighbor-joining clustering algorithm. The clustering results were displayed as a phylogenetic tree in Fig. 6, with each sequence assigned to its corresponding CRP family, plant family and plant clade. Two major clusters were observed; the β-ginkgotides and β-ginkgotide-like peptides (highlighted in dark brown in Fig. 6A) were separated from other CRPs such as the 6C-hevein-like peptides, CKAIs and cyclotides. As shown in Fig. 6B and C, the β-ginkgotides and β-ginkgotide-like peptides are found only in gymnosperms and are distributed among six families, Pinaceae, Podocarpaceae, Taxaceae, Cupressaceae, Gnetaceae and Welwitschiaceae. In contrast, all the other 6C-CRPs are found in both monocotyledons and dicotyledons of angiosperms such as Poaceae, Solanaceae and Fabaceae.

**Figure 7:**
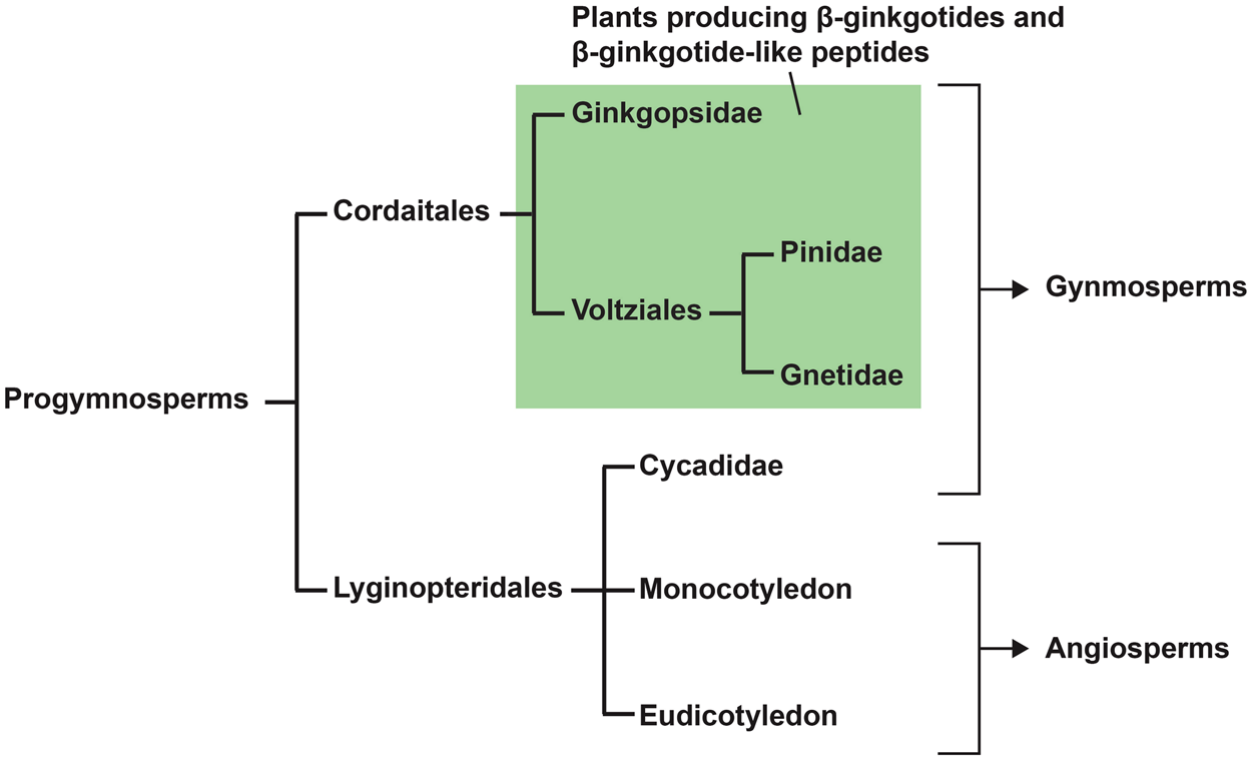
Occurrence of β-ginkgotide-like peptides *in planta*. Modern seed plants were evolved from a common ancestor progymnosperms and were separated into two major clades, Cordaitales and Lyginopteridales. Ginkgotide bL1 and bL1-like peptides were distributed only in Ginkgopsidae, Gnetidae and Pinidae (highlighted in green) but absent in Cycadidae and angiosperms.

Figure 7 illustrates the evolution of modern seed plants based on fossil records and genetic analyses^31, 32^. Christenhusz *et al*. classified gymnosperms into four distinct subclasses, including Ginkgoidae, Gnetidae, Pinidae and Cycadidae^33^. Modern seed plants were evolved from a common ancestor, the progymnosperms, and were separated into two major clades, Cordaitales and Lyginopteridales, in the Carboniferous period 354 million years ago. Cordaitales later evolved into Ginkgopsida, Pinopsida and Gnetopsida, whereas Cycadopsida and angiosperms evolved from Lyginopteridales^34^. Data mining showed that the β-ginkgotides and β-ginkgotide-like peptides are distributed only in the Ginkgoidae, Gnetidae and Pinidae but are absent in the Cycadidae and the angiosperms, suggesting that these compounds may have potentially originated from the same ancestor, Cordaitales^35, 36^. These results are in agreement with the previously described genetic analyses^37^ and provide an explanation for the absence of the β-ginkgotide-like peptides in angiosperms. Taken together, the transcriptomic data mining and neighbor-joining clustering analysis demonstrated that the β-ginkgotides belong to a new family of CRPs, are distributed only in gymnosperms and are absent in angiosperms.

### β-Ginkgotide Biosynthesis

**Figure 8:**
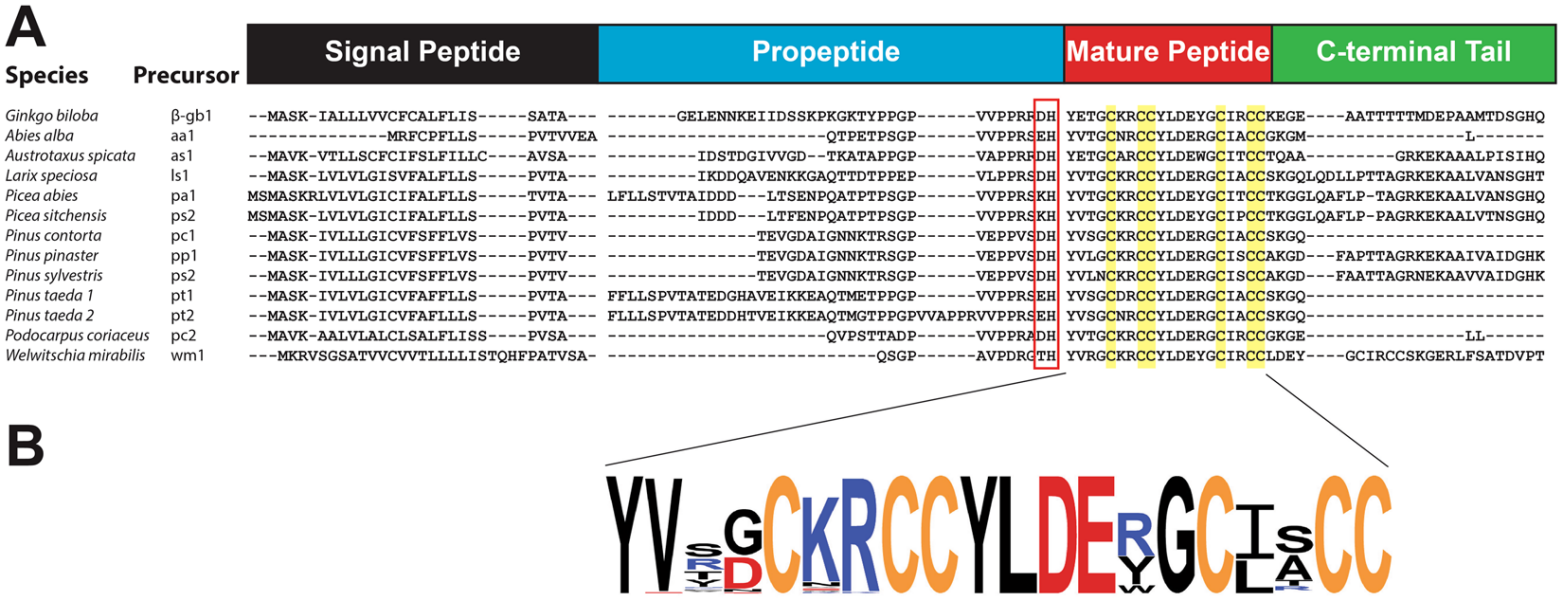
Precursor sequences alignment of β-ginkgotide *β*-*gb1* and twelve selected β-ginkgotide-like peptides. (**A**) The precursors are divided into four major domains, including a signal peptide, a propeptide, a mature peptide and a C-terminal tail. (**B**) The mature peptides of 76 β-ginkgotide-like peptides were summarized as a Weblogo. The cysteine, positive and negative charged residues were highlighted with yellow, blue and red, respectively.

Figure 8A displays the selected precursor sequences of the β-ginkgotides and β-ginkgotide-like peptides, CKAIs, 6C-hevein-like peptides, carboxypeptidase inhibitors cyclotides, thionins and jasmintides. The β-ginkgotides and β-ginkgotide-like peptides shared the same four-domain architecture that includes an endoplasmic reticulum signal peptide, a propeptide, a mature peptide and a C-terminal tail. The presence of the signal peptides suggests that they are secretory peptides, similar to the case in other known CRPs^3–5, 11, 38^. The release of the mature peptide involves the removal of the signal peptide by a signal peptidase and the cleavage of the propeptide and the C-terminal tail by an endopeptidase. The cleavage site between the propeptide and the mature peptide, where the peptide bond between the His and the Tyr was cleaved, was highly conserved. Subsequently, the C-terminal tail was removed, and the mature peptide was released for further post-translational modification in the Golgi apparatus and to be packed into vesicles for secretion.

Figure 8B illustrates the sequence logo that was obtained from the aligned sequences of the β-gB1 and 76 β-gB1-like peptides. The overall height of a stack represents the sequence conservation, while the height of each symbol within a stack represents the relative frequency with which the amino acid occurred^39^. Loop 2 was the longest, with six amino acids; five of these were highly conserved including Tyr10, Leu11, Asp12, Glu13 and Gly15. Both loops 1 and 3 comprised two amino acids. Interestingly, the majority of positive residues (Lys and Arg) were located in loop 2, whereas the negative residues (Asp and Glu) were located in loop 3.

**Figure 9:**
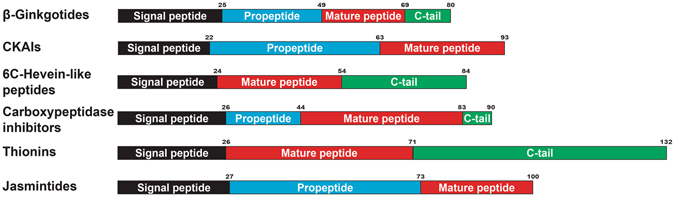
Summary of precursor sequences of β-gB1-like peptides and other known CRPs with six cysteine residues. The number on top of each domain represents the average number of amino acids.

A distinguishing characteristic of the β-ginkgotides and β-ginkgotide-like peptides, compared with other CRPs, was their relatively short full precursor sequence (80 aa) (Fig. 9). The β-ginkgotide and β-ginkgotide-like peptides have a relative longer C-tail (range: 2‒29 aa; mean: 11 aa) than those of carboxypeptidase inhibitors (range: 7 aa; mean: 7 aa) and CKAIs (no tail), but the lengths are similar to those of the 6C-hevein-like peptides (range: 29‒30 aa; mean 29.7 aa). Furthermore, the N-terminal repeated region in the cyclotides was absent in the β-ginkgotides and their homologs. These results suggest that the precursor sequence arrangement is conserved among β-ginkgotides and β-ginkgotide-like peptides but is significantly different in other CRPs. This knowledge of the β-ginkgotide and β-ginkgotide-like peptide precursors provides insight into their biosynthesis and is beneficial in developing a bacterial expression system and transgenic crops in the near future.

### Stability of β-Ginkgotide

To assess whether β-gB1 is relevant as a potential active component in traditional medicine, stability assays that simulated a decoction preparation process and mimicked a digestive system were employed and were illustrated in Figure S5, Supporting information. UPLC and MS data revealed that more than 75 and 90% of the β-gB1 remained intact after being boiled in water at 100 °C and incubated under acidic conditions at 37 °C for 1 hr, respectively. Likewise, β-gB1 was resistant to the endopeptidase trypsin and the exopeptidase carboxypeptidase A for up to 6 hr, with >90% of the peptide remaining.

### Cytotoxicity, Hemolytic and Antibacterial Effects of β-Ginkgotide

β-gB1 was used as a representative example to examine its antimicrobial, hemolytic and cytotoxic activities. Radial diffusion assays were used to determine the effect of β-gB1 on human pathogenic fungi and bacteria. No significant inhibitory effect of β-gB1was observed against *Escherichia coli*, *Candida albicans* and *Candida tropicalis* at concentrations up to 100 µM. Similarly, no hemolytic effect of β-gB1 was observed against erythrocytes obtained from human type O blood at concentrations up to 100 µM. Lastly, Vero and BHK cell lines were used for studying the *in vitro* cytotoxic effect that was exerted by β-gB1. The peptide was found to be nontoxic to both cell lines at concentrations up to 100 µM. Together with its high stability against harsh treatments, these results suggest that β-gB1 is nontoxic and can be used as a scaffold for peptide engineering.

## Conclusion

This study describes the discovery, characterization and synthesis of a new CRP family, the β-ginkgotides from *G*. *biloba*. The β-ginkgotides differ from other known families of CRPs by the following features: (1) short mature domain with 18‒20 amino acids and six cysteine residues, (2) unique cysteine spacing with CC motifs such as C‒CC‒C‒CC, (3) a novel disulfide connectivity of CysI‒IV, CysII‒VI and CysIII‒V, (4) a hyperdisulfide-constrained, compact structure with only loops and no regular secondary structure elements, and (5) they are gymnosperm-specific.

Data mining found an additional 76 β-ginkgotide-like peptides *in planta*. In addition, a clustering analysis confirmed that the β-ginkgotides and β-ginkgotide-like peptides represent the first-in-class hyperdisulfide-constrained peptide family. Overall, our discovery of this family expands the existing families of the CRPs in the chemical space of 2–6 kDa and provides new insights to their sequence diversity, cysteine arrangement, structure, biosynthesis, occurrences and distribution *in planta*.

## Materials and Methods

### Plant Materials

*G*. *biloba* seeds were purchased from a local herb distributor (Hung Soon Medical Trading Ltd., Singapore) and authenticated by an experienced traditional Chinese medicine practitioner from the Nanyang Technological University Traditional Chinese Medicine Clinic, Singapore. The authentication criteria were based on the macroscopic and microscopic characteristics described in the Pharmacopoeia of the People’s Republic of China^7, 40, 41^. A voucher sample (NTUH-SGB20161010-01) was deposited at the Nanyang Technological University Herbarium, School of Biological Sciences, Singapore.

### Purification of β-Ginkgotide

*G*. *biloba* seeds (2 kg) were homogenized in 20 L of MilliQ water and incubated for 2 hr at room temperature. The homogenate was centrifuged at 9000 rpm for 20 min, and the supernatant was loaded onto a reversed-phase flash column with 500 g of C_18_ powder (Grace, MD, USA) packed in a Büchner funnel (250 × 22 mm). The elution was carried out using increasing concentrations of ethanol (20, 40, 60 and 80% v/v). The 20 and 40% eluents were loaded onto a Sepharose Fast Flow SP (GE Healthcare Life Sciences, Little Chalfont, United Kingdom) flash column. The column was percolated with 20 mM potassium dihydrogen phosphate at a pH of 3.0 and eluted with 20 mM potassium dihydrogen phosphate with 1 M sodium chloride at a pH of 3.0. To obtain the purified peptides, multiple rounds of preparative reversed-phase- (RP-) and strong cation exchange-high-performance liquid chromatography (SCX-HPLC) were performed as previously described^5, 42^. The purification was performed on a Prominence UFLC system (Shimadzu, Kyoto, Japan) attached to an Aeris peptide XB-C_18_ column (Phenomenex, CA, USA; particle size 5 µm, 250 × 22 mm) or a polysulfoethyl A column (PolyLC, MA, USA; particle size 10 µm, 250 × 22 mm).

### β-Ginkgotide Sequence Determination

The β-ginkgotide primary amino acid sequence was determined as described previously^5^. Briefly, lyophilized β-ginkgotide (10 µg) was reconstituted in 30 µL of 20 mM dithiothreitol (DTT) and incubated at 37 °C for 1 hr. The reduced peptide was alkylated with 200 mM iodoacetamide at 37 °C for 1 hr in the dark, desalted using a C_18_ Zip-tip, and lyophilized. The S-alkylated β-ginkgotide was analyzed by an ABI 4800 matrix-assisted laser desorption/ionization time-of-flight mass spectrometer (MALDI-TOF MS) (Applied Biosystem, MA, USA). The sample (0.5 µL) was mixed with 0.5 µL of matrix consisting of a saturated solution of α-cyano-4-hydroxycinnamic acid in 50/50 (v/v) acetonitrile/water with 0.1% trifluoroacetic acid. The mixture was spotted onto a MALDI plate and dried at room temperature. The MALDI-TOF MS was operated in positive ion reflector mode, acquiring 2000 shots (20 positions per spot; 100 shots per position) per spectrum with a laser intensity at 4500. The accelerating and grid voltages were set at 20 and 16 kV, respectively. The data were acquired between 2000 and 5000 Da using AB 4000 Series Explorer v3.5.1 and were analyzed using Data Explorer v4.9 (Applied Biosystem, MA, USA). The primary sequence was obtained by interpreting the *b*- and *y*-ions formed during the tandem MS/MS fragmentation. The assignments of isobaric residues Ile/Leu and Lys/Gln were based on transcriptomic data obtained from the One thousand plants database (OneKP; accession: SGTW-2035618)^43^.

### Disulfide Mapping

β-Ginkgotide β-gB1 was partially reduced in 400 µL of 100 mM citrate buffer (pH 3.0) with 50 mM tris(2-carboxyethyl)phosphine at 37 °C for 17 min. Subsequently, 250 mM N-ethylmaleimide (NEM) was added and incubated at 37 °C for 1 hr. The reaction mixture with the partially alkylated peptide was monitored using MALDI-TOF MS and purified by RP-HPLC. The two- and four-NEM alkylated intermediate species were further reduced with 20 mM dithiothreitol at 37 °C for 1 hr and alkylated with 40 mM iodoacetamide at 37 °C for 1 hr. The fully alkylated β-gB1 was desalted and sequenced by MALDI-TOF/TOF MS/MS.

### Stability Assay

#### Heat

The β-ginkgotide β-gB1 (28 µg) was incubated in a heat block at 100 °C for 2 hr. At each time-point (0 and 1 hr), 20 µL of the treated sample was aliquoted and quenched in an ice bath for 10 min.

#### Acid

The β-ginkgotide β-gB1 (28 µg) was incubated in 1 M hydrochloride acid (pH 2.0) at room temperature for 2 hr. At each time-point (0 and 1 hr), 20 µL of the treated sample was aliquoted and quenched by adding 2 µL of 1 M sodium hydroxide.

#### Trypsin

The β-ginkgotide β-gB1 (28 µg) was added to 100 µL of 100 mM ammonium bicarbonate buffer (pH 7.8) and incubated in a water bath at 37 °C for 6 hr. At each time-point (0 and 6 hr), 20 µL of the treated sample was aliquoted and quenched by adding 5 µL of 1 M hydrochloric acid.

#### Carboxypeptidase A

The β-ginkgotide β-gB1 (28 µg) was added to a solution of 50 mM Tris-HCl (pH 7.5) and 100 mM sodium chloride with 100 nM carboxypeptidase B. The mixture was incubated in a water bath at 37 °C for 6 hr. At each time-point (0 and 6 hr), 20 µL of the treated sample was aliquoted and quenched by adding 5 µL of 1 M hydrochloric acid.

#### Analytical HPLC

The reaction mixture was analyzed by a Nexera UHPLC system (Shimadzu, Kyoto, Japan) equipped with an Aeris peptide XB-C_18_ column (Phenomenex, CA, USA; particle size 3.6 µm, 100 × 4.6 mm). The eluents were monitored with MALDI-TOF MS, and the area under the peak before and after the treatment was determined to evaluate stability.

### Synthesis and Oxidative Folding of β-Ginkgotide

β-Ginkgotide β-gB1, obtained from GL Biochem (Shanghai, China), was synthesized by solid phase peptide synthesis using Fmoc (N-(9-fluorenyl)methoxycarbonyl) chemistry and purified by C18-reversed-phase HPLC to 85% purity. Optimization of the folding conditions was performed by varying the concentrations of co-solvent and redox reagents as well as the duration and was summarized in Table S1 (Supporting information). The final peptide concentration was 42.12 μM. After 2 hr, the reactions were quenched by acidification with 2 M hydrochloric acid, and the products were profiled using RP-UPLC. Native β-gB1 that was purified from the plant was used as a standard to monitor the folding process. The yield was determined by comparing the area under the peak of β-gB1 before and after the folding reaction.

### NMR Structure Determination

2D NOESY and TOCSY experiments were used to achieve an unambiguous peak assignment and determine the structure of β-gB1. All the NMR spectra were acquired on a 600 MHz NMR spectrometer (Bruker, IL, USA) equipped with a cryogenic probe. The temperature of the NMR experiment was 298 K. The protein concentration of the β-gB1 sample was 1 mM and contained 5% D_2_O and 95% H_2_O (pH 3.5). The mixing times were 80 ms and 200 ms for TOCSY and NOESY respectively. The spectrum width was set to 12 ppm with the ^1^H carrier frequency set at 4.700 ppm. The spectra were processed using NMRPipe software^44^. The NOE cross-peaks were assigned using Sparky software on the 2D NOESY and TOCSY spectra^45^. The structure determination was performed using the method of simulated annealing as defined by the script of the CNSsolve 1.3 software^46^. The distance restraints were loaded for the structure calculation and were first divided into three classes based on the intensities of the NOE peaks: strong, 0 < d ≤ 1.8 Å; medium, 1.8 < d ≤ 3.4 Å; and weak, 3.4 < d ≤ 5 Å. Dihedral angle restraints were determined based on the J coupling splitting, J_HN-Hα_, in the 1D-NMR spectrum. The ϕ angle was considered to be between −100 and −160° when the splitting was more than 8 Hz. The structure was verified using the PROCHECK program^47^ and displayed using Chimera version 1.6.2^48^ or Pymol version 1.8^49^. The NMR structure of β-gB1 was deposited in the Protein Data Bank with an accession number of 5XIV with a BMRB number of 36079.

### Biological Assay

#### Cytotoxicity

Human umbilical vein endothelial cells (HUVECs) were seeded onto 96-well plates in DMEM medium (10% FBS and 1% penicillin and streptomycin) and grown at 37 °C with a 5% CO_2_ atmosphere until confluence. After 24 hr, various concentrations of β-ginkgotide β-gB1 (1–100 µM) were added, and the plates were incubated for 24 hr. The cell viability was assessed by 3-(4,5-dimethylthiazol-2-yl)-2,5-diphenyltetrazolium (MTT) assay. Triton X-100 solution (1%) was used as a positive control^4^.

#### Hemolytic effect

Erythrocytes were isolated from fresh blood type O+ by centrifugation for 15 min at 10000 rpm. The erythrocytes were diluted with phosphate-buffered saline, mixed with various concentrations of β-ginkgotide β-gB1 (1–100 µM) and incubated for 4 hr at 37 °C. The hemolytic effect was examined using a microplate reader set at 415 nm^3^.

#### Antibacterial effect

Radial diffusion assays were performed on gram-negative *Escherichia coli* (ATCC 25922) and gram-positive *Staphylococcus aureus* (ATCC 12600) as described previously^11, 38^.

### Data Mining and Bioinformatics Analysis

The transcriptomic database search was performed on public domains GenBank and OneKP^30^. The cleavage site of the signal peptide in the precursor sequence was determined by SignalP 4.0^50^. The primary amino acid sequence and precursor sequence alignments were performed using Kalign^51^. Neighbor-joining clustering analysis was performed on MEGA 6.0^52^, and a phylogenetic tree was constructed using a bootstrap test of 1000 replicates. The phylogenetic tree was displayed using iTOL^53^. The sequence logo were generated with WebLogo^39^. The pairwise structural alignment was performed using TM-align algorithm^54^.

## Electronic supplementary material

Supplementary Information
