# Supplementary material for: β-Ginkgotides: Hyperdisulfide-constrained peptides from Ginkgo biloba
Source: Sci Rep. 2017 Jul 21;7:6140. doi: 10.1038/s41598-017-06598-x (PMC5522442; doi:10.1038/s41598-017-06598-x)
Supplement: Supplementary file 1 — Supplementary Information [file 41598_2017_6598_MOESM1_ESM.doc]

**β-Ginkgotides: Hyperdisulfide-constrained peptides from *Ginkgo biloba***

Ka H. Wong^1^, Wei Liang Tan^1^, Tianshu Xiao^1^, James P. Tam^1*^

^1^School of Biological Sciences, Nanyang Technological University, Singapore 637551

**Correspondence:**

* Author to whom correspondence should be addressed:

Prof James P Tam

60 Nanyang Drive,

School of Biological Sciences,

Nanyang Technological University, Singapore, 637551

E-mail: jptam@ntu.edu.sg

Tel: + 65 63162863


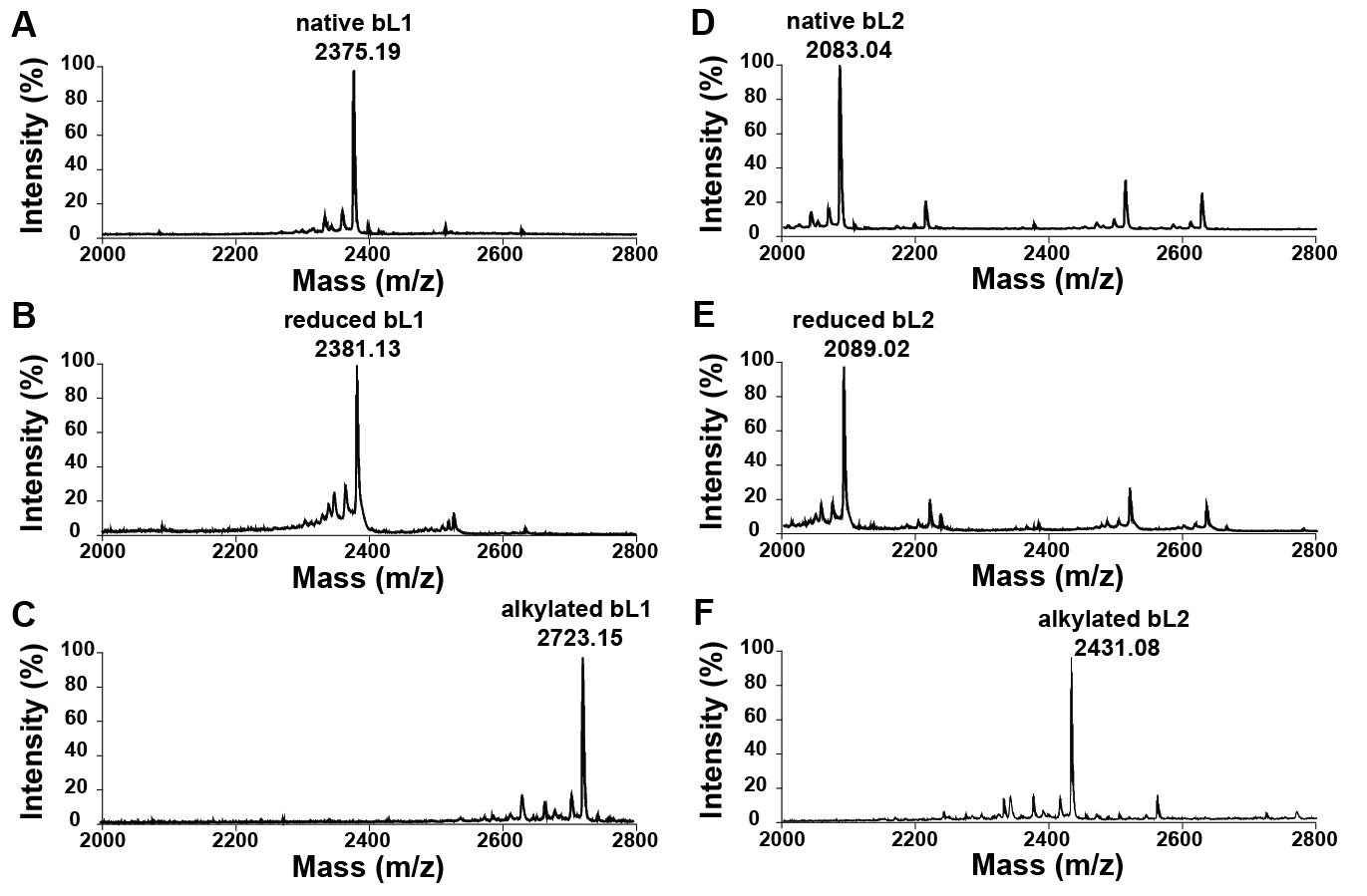


Figure S1. The MALDI-TOF spectra of ginkgotides. The native β-ginkgotide β-gB1 and β-gB2 (A & D) were S-reduced by dithiothreitol (B & E) and S-alkylated by iodoacetamide. The mass difference before and after the reductive S-alkylation of β-ginkgotide β-gB1 and β-gB2 were monitored using MALDI-TOF MS. Each S-alkylated Cys caused a mass increase of 58 Da. A mass shift of 348 Da (C & F) suggests the presence of six Cys in each peptide.


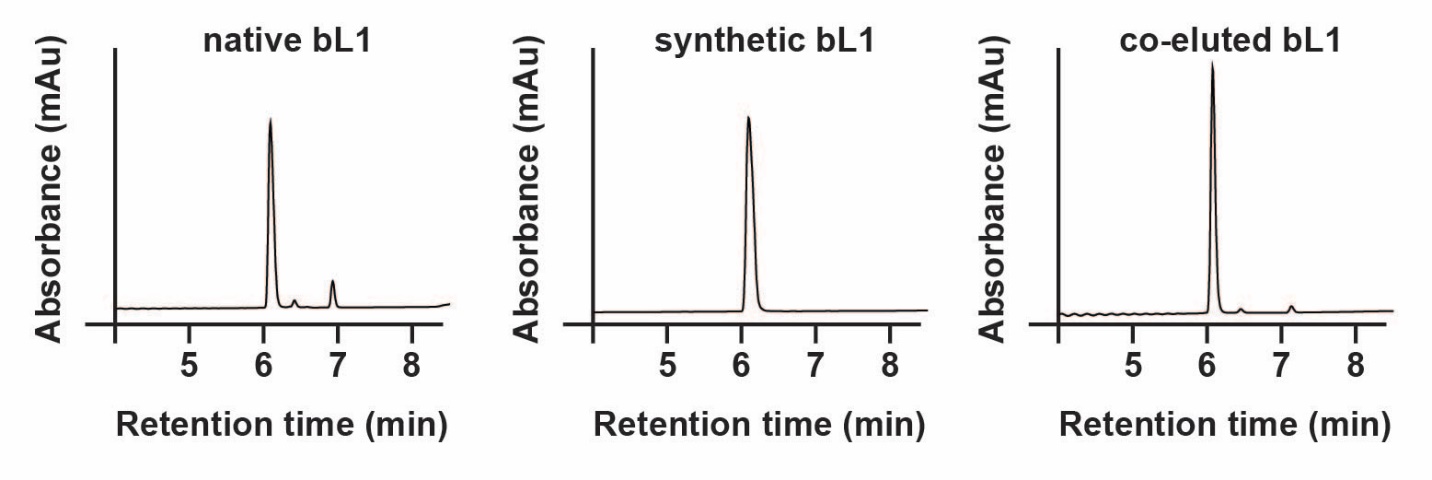


Figure S2. Chromatograms of the native and synthetic β-gB1.


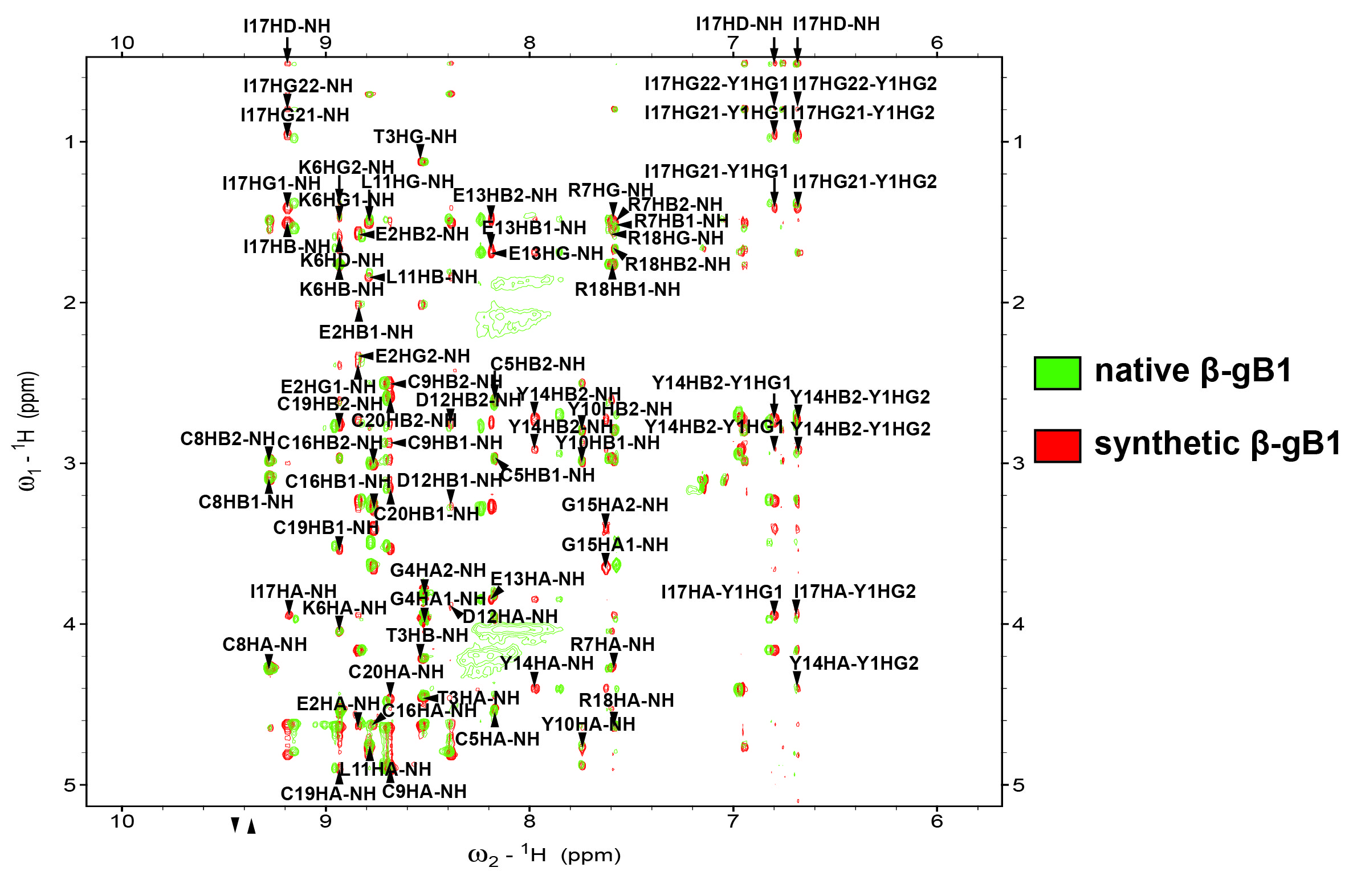


Figure S3. Chemical shift assignment of β-gB1NOESY spectrum of the NOE cross peaks between side chain protons and amide protons.


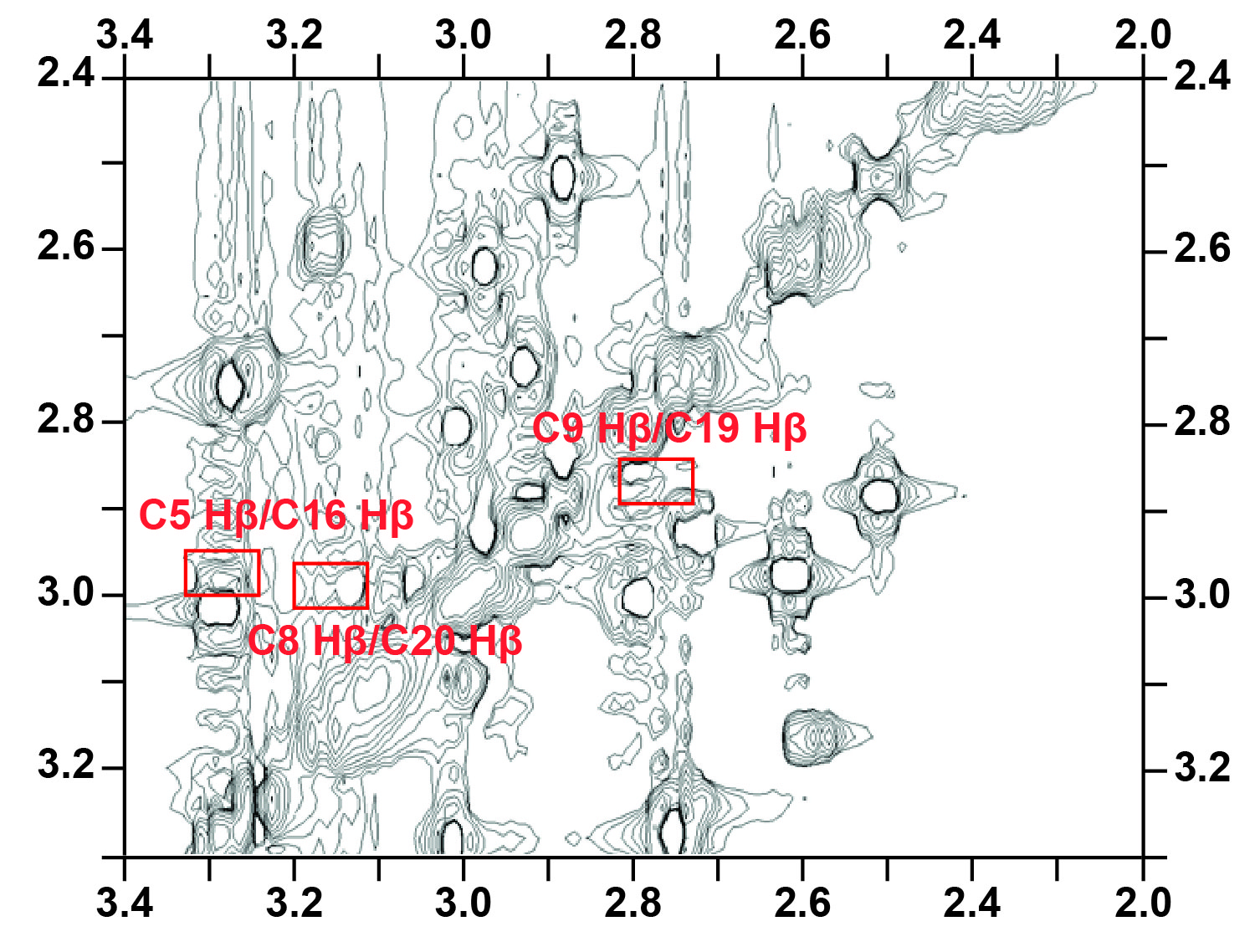


Figure S4. NOE cross peak between the Hβs of the each disulfide bond in β-gB1.


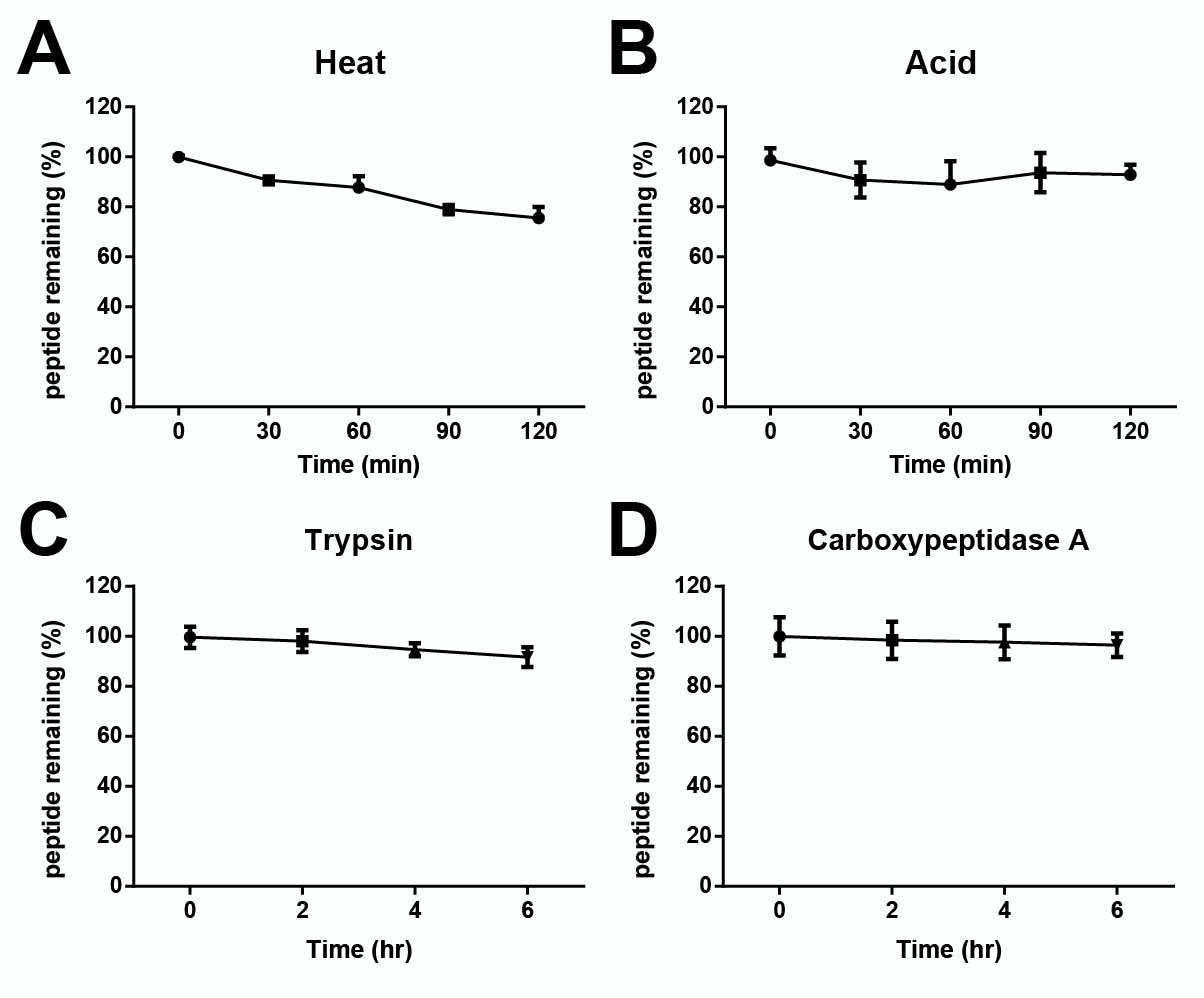


Figure S5. Stability assays of β-gB1. (A) Thermal stability of β-gB1 incubated at 100^o^C for 120 minutes. (B) Acidic condition stability of bL1 incubated in 0.2 M HCl (pH 2.0) at 37^o^C for 120 minutes. Enzymatic stability of bL1 against (C) trypsin and (D) carboxypeptidase A, respectively for 6 hours in buffer as suggest by manufactures at 37^o^C. The molecular weight of the peak was determined by MALDI-TOF MS.

Table S1. Parameters of the oxidative folding condition.

| **Run** | **GSH (mM)** | **GSSG (mM)** | **Cystamine (mM)** | **Cysteine amine (mM)** | **DMSO (%)** | **iPrOH (%)** | **Time (hr)** | **Yield (%)** |
| --- | --- | --- | --- | --- | --- | --- | --- | --- |
| **1** |  |  | 400 | 4 |  |  | 24 | 28.9 |
| **2** | 4 | 400 |  |  |  |  | 24 | 36.6 |
| **3** |  |  | 400 | 4 | 10 |  | 24 | 38.8 |
| **4** | 4 | 400 |  |  | 10 |  | 24 | 55.3 |
| **5** |  |  | 400 | 4 |  | 20 | 24 | 17.4 |
| **6** | 4 | 400 |  |  |  | 20 | 24 | 27.6 |
| **7** | 4 | 800 |  |  | 10 |  | 2 | 66.0 |
| **8** | 4 | 800 |  |  | 10 |  | 6 | 65.4 |
| **9** | 4 | 800 |  |  | 10 |  | 24 | 65.1 |
| **10** | 2 | 400 |  |  | 10 |  | 2 | 76.7 |
| **12** | 2 | 400 |  |  | 10 |  | 6 | 73.9 |
| **13** | 2 | 400 |  |  | 10 |  | 24 | 73.4 |

GSH: glutathione; GSSH: glutathione disulfide; DMSO: dimethyl sulfoxide; iPrOH: isopropanol. The reaction was incubation at room temperature.

Table S2. NMR experimental and structural statistics of synthetic β-gB1.

| **NMR Distance Restraints** |  |
| --- | --- |
| Intra-Residue NOE (|i-j|=0) | 73 |
| Sequential NOE(|i-j|=1) | 62 |
| Medium-Range NOE (1<|i-j|≤5) | 19 |
| Long-Range NOE (|i-j|>5) | 52 |
| All | 206 |
| Hydrogen Bonds | 9 |
| **Structural Statistics (20 residues, Y1-C20)** |  |
| NOE Violation | 0.034 ± 0.001 Å |
| Maximum NOE Violation | 0.037 Å |
| Ramachandran Plot Region (20 residues) |  |
| Residues in Most Favored Regions | 8 (50.0%) |
| Residues in Additional Allowed Regions | 4 (25.0%) |
| Residues in Generously Allowed Regions | 4 (25.0%) |
| Residues in Disallowed Regions | 0 (0%) |
| Number of End-Residues (excl. Gly and Pro) | 2 |
| Number of Glycine Residues | 2 |
| Number of Proline Residues | 0 |
| **Mean RMSD from the Average Coordinates (20 residues, Y1-C20)** | |
| Backbone Atoms | 0.74 ± 0.30 |
| Heavy Atoms | 1.77 ± 0.44 |

Experimental Restraints and Structural Statistics of 20 Lowest-Energy Structures of β-gB1 among the 100 Structures Generated by CNSsolve 1.3.

Table S3. NMR restraints and statistics of the solution structure of β-gB1 generated by CNSsolve 1.3.

|  | **HN (ppm)** | **Hα (ppm)** | **Hβ (ppm)** | | **Others (ppm)** |
| --- | --- | --- | --- | --- | --- |
| Y1 |  | 4.171 | 3.241 | 2.74 | Hδ, 6.833, 6.725 |
| E2 | 8.878 | 4.635 | 2.026 | 1.578 | Hγ, 2.395, 2.345 |
| T3 | 8.575 | 4.461 | 4.221 |  | Mγ, 1.131 |
| G4 | 8.559 | 3.968, 3.821 |  |  |  |
| C5 | 8.207 | 4.534 | 2.975 | 2.616 |  |
| K6 | 8.97 | 4.051 | 1.771 |  | Hγ, 1.474, 1.39; Qδ, 1.624 |
| R7 | 7.631 | 4.274 | 1.567 | 1.555 | Qγ, 1.496; Qδ, 7.075 |
| C8 | 9.313 | 4.654 | 3.104 | 2.989 |  |
| C9 | 8.726 | 4.888 | 2.876 | 2.509 |  |
| Y10 | 7.776 | 4.775 | 2.998 | 2.803 | Hδ, 6.981, 6.792 |
| L11 | 8.824 | 4.824 | 1.848 |  | Hγ, 1.512; Mδ1, 0.712 |
| D12 | 8.422 | 3.899 | 3.276 | 2.76 |  |
| E13 | 8.225 | 3.855 | 1.693 | 1.488 | Qγ, 1.695 |
| Y14 | 8.009 | 4.406 | 2.921 | 2.734 | Hδ, 6.998, 6.718 |
| G15 | 7.662 | 3.644, 3.415 |  |  |  |
| C16 | 8.802 | 4.631 | 3.288 | 3.012 |  |
| I17 | 9.224 | 3.95 | 1.519 |  | Hγ1, 1.42; Hγ2, 0.964, 0.804; Mδ, 0.519 |
| R18 | 7.624 | 4.645 | 1.776 | 1.676 | Qγ, 1.605; Qδ, 7.179 |
| C19 | 8.969 | 4.91 | 3.541 | 2.767 |  |
| C20 | 8.722 | 4.471 | 3.164 | 2.586 |  |

Table S4. Lowest Energy Analysis of β-gB1.

| **Pattern** | **Disulfide Bond Pattern** | **Energy (kcal/mol)** |
| --- | --- | --- |
| **1** | Cys5-Cys16, Cys8-Cys20, Cys9-Cys19 | 357.546±5.558 |
| **2** | Cys5-Cys16, Cys8-Cys19, Cys9-Cys20 | 727.117±178.714 |
| **3** | Cys5-Cys16, Cys8-Cys9, Cys19-Cys20 | 753.001±14.461 |
| **4** | Cys5-Cys9, Cys8-Cys20, Cys16-Cys19 | 728.265±62.364 |
| **5** | Cys5-Cys19, Cys8-Cys20, Cys16-Cys9 | 516.167±37.634 |
| **6** | Cys5-Cys9, Cys8-Cys16, Cys20-Cys19 | 686.440±40.376 |
| **7** | Cys5-Cys9, Cys8-Cys19, Cys16-Cys20 | 617.512±8.374 |
| **8** | Cys5-Cys19, Cys8-Cys9, Cys16-Cys20 | 567.774±47.824 |
| **9** | Cys5-Cys19, Cys8-Cys16, Cys9-Cys20 | 471.874±17.720 |
| **10** | Cys5-Cys8, Cys16-Cys20, Cys9-Cys19 | 1004.411±62.544 |
| **11** | Cys5-Cys8, Cys16-Cys9, Cys20-Cys19 | 693.639±66.826 |
| **12** | Cys5-Cys8, Cys16-Cys19, Cys9-Cys20 | 609.871±21.934 |
| **13** | Cys5-Cys20, Cys8-Cys16, Cys9-Cys19 | 673.339±64.825 |
| **14** | Cys5-Cys20, Cys8-Cys9, Cys16-Cys19 | 599.741±26.316 |
| **15** | Cys5-Cys20, Cys8-Cys19, Cys9-Cys16 | 466.585±10.692 |

Fifteen combinations of disulfide bonds were generated and assumed for structure calculation. The averaged value of the overall energies of the 20 structures with the lowest energies among 100 structures is listed in the last column for each disulfide bond pattern.

Table S5. TM align score between β-ginkgotide β-gB1 and other CRPs with 6 cysteine residues.

| **CRP family** | **Peptide** | **PDB** | **TM align score** |
| --- | --- | --- | --- |
| CKAI | AAI | 1QFD | 0.11733 |
|  | aL1 | 2MI9 | 0.20559 |
|  | wR1 | 2MAU | 0.11184 |
| Carboxypeptidase inhibitor | PCI | 1H20 | 0.13530 |
| 6C-Hevein-like peptide | aSG1 | N.A. | 0.14651 |
|  | Ac-AMP | 1MMC | 0.21019 |
|  | Sm-AMP-1 | 2KUS | 0.13949 |
|  | Sm-AMP-2 | 2N1S | 0.14474 |
| Thionin | Crambin | 3U7T | 0.12664 |
|  | gamma 1H | 1GPT | 0.11754 |
|  | hellethionin D | 3SZS | 0.17490 |
|  | NaD1 | 1MR4 | 0.16882 |
|  | Viscotoxin B | 1JMP | 0.11550 |
| Jasmintide | jS1 | 2N5Q | 0.18266 |

Pairwise alignment using β-ginkgotide β-gB1 was performed. N.A.: Not available. The structure of aSG1 was kindly provided by the authors. A TM-score value <0.3 means that the two candidates have random structural similarity, while a value >0.5 means that they shared the same protein fold.

Table S6. Sequences of β-ginkgotide-like peptides.

| **Species** | **Accession number** | **Full sequence** |
| --- | --- | --- |
| *Abies alba* | HE620364.1 | MRFCPFLLSPVTVVEAQTPETPSGPVVPPRSEHYVTGCNRCCYLDERGCIACCGKGML |
| *Abies alba* | AM169090.1 | MASKLVLVLGICVFSLFLLSPPVTAIESPPVTAIQDAEPVVPPRSDHYVSGCKRCCYLDERGCIACCSKGHVLQDLLPMTAGRKEKAAVIANSGHK |
| *Abies lasiocarpa* | VSRH-2050857 | MASKAVLVLGLCVFALFLLSPVTVVEAQTPETPSGPVVPPRSEHYVTGCKRCCYLDERGCIACCGKGML |
| *Amentotaxus argotaenia* | IAJW-2014198 | MAPKIEVVFVLCIFTLCLLQSSPSAAAQVTETPPEGPVAPPWQKHYVRDCKRCCYLDEWGCLSCCGKGGQI |
| *Amentotaxus argotaenia* | IAJW-2009697 | MASKIAVVFALCIFTLCLLHSSPSATAQVTETPPEGPVVPPREKHYVRDCKRCCYLDEWGCLSCCGKGNQL |
| *Athrotaxis cupressoides* | XIRK-2054579 | MASKVVMFFTLCVFAVFLLQSSTSATATDAIFGENKAHEEQVAPDPQKHYVYDCKRCCYLDEYGCISCCGKGQQL |
| *Austrocedrus chilensis* | YYPE-2061402 | MASKAVIVFTLCVFAVFLLQSPSVSATATAALFGENKAQQDQVVPDRQKHYVYDCKRCCYLDEYGCLSCCGKGQQL |
| *Austrotaxus spicata* | BTTS-2069273 | MASKIAMVFAVCIFALCLLQSSPSATVTAQVTETPPQGPVAPPLQKHYVRDCKRCCYLDEWGCLSCCGKAGQI |
| *Callitris gracilis-March* | IFLI-2130165 | MASKAVMVFALCFSAVFLLQSSKVSATATDVLFRENKAEIGQPGVVVPDREKHYVYNCKRCCYLDEYGCLSCCGKGQQH |
| *Callitris macleayana-March* | RMMV-2044903 | MASKAVMVFALCFLAVFLLPSSKVSATATDVLFGENKAQIEQPEVVVPDREKHYVYNCKRCCYLDEYGCLSCCGKGQQL |
| *Calocedrus decurrens* | FRPM-2047082 | MASKAVMVFTLCVFAVFLLQSQTSATAIDALFGESKAQEGPAVPDLQKHYVYDCKRCCYLDEYGCLSCCGKGQ |
| *Cathaya agryrophylla* | NPRL-2111073 | MASKLVLVLCICIFALFLLSPVRAIEDEHAVENQKEAQTTDTPPEPVVPPRSDHYVTGCKRCCYLDERGCIACCSKGQL |
| *Cedrus atlantica* | SRR065007.272056.2 | MIMTVEIKKEAQTTEPPSGPVLPPRSEHYVTGCKRCCYLDERGCIACCSKGQ |
| *Cedrus libani* | GGEA-2065693 | MASKLVLVLGICVFALYLLSPVTAIEDDHAVEIKKEAQTTEPPSGPVLPPRSEHYVTGCKRCCYLDERGCIACCSKGQ |
| *Cunninghamia lanceolata* | OUOI-2046637 | MASKVVMVITLCVFAVFLLQSSTSATAIDALLGENKAQEGPVVPDRQKHYVKDCKRCCYLDEYGCITCCGKGEQL |
| *Cupressus dupreziana* | QNGJ-2061402 | MASKAVMVFTLCIFAVFLLQSQTSATAIDALFGVSKAQQGPAVPDLQKHYVRECKRCCYLDEYGCLSCCGKGQQL |
| *Dacrycarpus compactus* | FMWZ-2007957 | MAVPVKAALVLALCLSALFLISPSVSAEVPSTPSEQVVPPRADHYVSGCKRCCYLDERGCITCCGKGEQ |
| *Dacrydium balansae* | IZGN-2010597 | MAVKVALVLVLCFSALFLTSSPVSAQDPSTTPPGLVVPPVADHYVSGCKRCCYLDERGCIACCGKGQ |
| *Diselma archeri* | GKCZ-2003597 | MASKAIKLFTLCVFALFFLQASSVSDTSIDALFGENKAQIEQTAPDPGKHYVHDCKRCCYLDEWGCLSCCGKGQQL |
| *Diselma archeri* | GKCZ-2003596 | MASKAVKLFTLCVFALFFLQSSSVSATSIDALFGENKAQIEQTAPDPGKHYVHDCKRCCYLDEWGCLSCCGKGQQL |
| *Fokienia hodginsii* | UEVI-2052355 | MASKAVMVFTLCVFAVFLLQSSTSATAIDALFGENKAREGPVFPDLQKHYVYDCKRCCYLDEYGCLSCCGKGQQL |
| *Gnetum gnemon* | SRR064399.62918.2 | MKKARSEVVTMCFVALLLFCIVVDLQVVSVPFASARSGPVVPDRGTHYVSGCQRCCYLDEYGCIRCCGKGGA |
| *Juniperus scopulorum* | XMGP-2052052 | MASKAVMVFTLCVFAVFLFQSQTSATAIDALFGVSKAQQWPVFPELQKHYVYDCKRCCYLDEYGCLSCCGKGQQL |
| *Keteleeria evelyniana* | JUWL-2015067 | MASKVVLVLGICVFALFLLSPVTAIEDDHAVEIKTEAQTMQTPSGPVVPPLSEHYVTGCKRCCYLDERGCIACCGKGQL |
| *Keteleeria evelyniana* | JUWL-2006177 | MASKVVLVLGTCVFALFLLSPVTAVEEDHAVEIKKEAQTTDNPPVVVPDRSEHYVRGCKRCCYLDERGCIACCGKGYLLPGTAGRKEKTALVANSGQQ |
| *Larix speciosa* | WVWN-2048240 | MASKLVLVLGISVFALFLLSPVTAIKDDQAVENKKGAQTTDTPPEPVLPPRSDHYVTGCKRCCYLDERGCIACCSKGQLQDLLPTTAGRKEKAALVANSGHT |
| *Metasequoia glyptostroboides* | NRXL-2001551 | MASKVVMVFTLCVFAVFLLQSSTSATATDALLGENKAQEGPVVPDLQKHYVRDCKRCCYLDEYGCLSCCGKGQQL |
| *Microbiota decussata* | XQSG-2003025 | IVLTLCIFAVFLLQSSTSATATDALLGENKPQEGPVVPDRQKHYVRDCKRCCYLDEYGCLSCCGKGQQL |
| *Microbiota decussata* | XQSG-2003023 | MASKAVMVFTLCVFAVFLLQSSTSATAIDALFGESKAQEGPVAPDLQKHYVYDCKRCCYLDEYGCLSCCGKGEQL |
| *Microcachrys tetragona* | MHGD-2010646 | MAVKAALVLALCLSALFLVSSPVSAQVPSTPPGPVVPPRSDHYVSGCKRCCYLDERGCIQCCGKGQLL |
| *Microstrobos fitzgeraldii* | BBDD-2064059 | MAVKVALVLALCVSALFLVSSPVSAQVPSPTPPGPVLPPPAEHYVSDCKRCCYLDERGCIRCCGKGE |
| *Nageia nagi* | UUJS-2001299 | MAVKAALVLALCLSALFLISSPVSAQVPSPTPPEPVVPPRADHYVSGCRRCCYLDERGCIRCCGKGEQ |
| *Neocallitropsis pancheri* | JDQB-2007642 | MASKALMLFTICVLALFLLESSAVSATATDALYGENKAQIEQPKVVVPDREKHYVYDCKRCCYLDEYGCLSCCGKGQQL |
| *Nothotsuga longibracteata* | AREG-2061186 | MASNVVLVLGICFFALFMLSPVTAIEDDHAVEFKKEAQTTPPPPPPYEPVLPPRSEHYVVGCKRCCYLDERGCIACCSKGGLQDLLPMTAGRKEKAAVVANSGHQ |
| *Papuacedrus papuana* | OVIJ-2078173 | MASKAVMVFTLCVFAVFLLQSASVSATATDALFGENKAHEEQVVPDRQKHYVYDCKRCCYLDEYGCLSCCGKGEQL |
| *Parasitaxus usta* | JZVE-2032566 | MAVKAAVVLAVCLSALFLISSPVSAQDPSTPPGVVIPPRADHYVSGCKRCCYLDERGCIECCGKGMLL |
| *Picea abies* | AM168782.1 | MSMASKRLVLVLGICIFALFLLSTVTAIDDDLTSENPQATPTPSGPVVPPRSKHYVTGCKRCCYLDEYGCITCCTKGGLQAFLPTAGRKEKAALVANSGHQ |
| *Picea abies* | AM168533.1 | MASKLVLVLGICVFSLFLLSPPVTAIESPPVTAIQDDEPVVPPRSDHYVSGCKRCCYLDERGCIACCSKGHVLQDLLPMTAGRKEKAAVIANSGHK |
| *Picea engelmannii x Picea glauca* | CO203618.1 | MASKLVLVLGICVFSLFLLSPPVTAIESPPVTAIQDEPVVPPRSDHYVSGCKRCCYLDERGCIACCSKGELLQDLLPMTAGRKEKAAVIANSGHK |
| *Picea glauca* | EX308427.1 | MSMASKLVLVLGICIFALFLLSPVTAIDDDLTSENPQATPTPSGPVVPPRSKHYVTGCKRCCYLDEYGCITCCTKGGLLVERKKQH |
| *Picea glauca* | CO488051.2 | MASKLVLVLGICVFSLFLLSPPVTAIESPPVTAIQDDEPVVPPRSDHYVSGCKRCCYLDERGCIACCSKGELLQDLLPMTAGRKEKAAVIANSGHK |
| *Picea glauca* | DR553067.1 | MASKLVLVLGICVFSLFLLSPPVTGIAIQDDEPVVPPRSDHYVSGCKRCCYLDERGCIACCSKGHVLQDLLPMTAGRKEKAAVIANSGH |
| *Picea sitchensis* | CO216005.1 | MASKLVLVLGICVFSLFLLSPPVTGIAIQDDEPVVPPRSDHYVSGCKRCCYLDERGCIACCSKGHVLQDLLPMTAGRKEKAAVIANSGHK |
| *Picea sitchensis* | DR543902.1 | MSMASKLVLVLGICIFALFLLSPVTAIDDDLTFENPQATPTPSGPVVPPRSKHYVTGCKRCCYLDEYGCIPCCTKGGLQAFLPPAGRKEKAALVTNSGHQ |
| *Picea engelmanii* | AWQB-2049927 | MASKLVLVLGICVFSLFLLSPPVTAIESPPVTAIQDDEPVVPPRSDHYVSGCKRCCYLDERGCIACCSKGQLLQDLLPMTAGRKEKAAVIANSGHK |
| *Pilgerodendron uviferum* | ETCJ-2003441 | MASKVAMLFTLSVFSVFLLQSSSLSVTATDALFLFVGNKAQPEQAVPDRQKHYVYDCKRCCYLDEYGCLSCCGK |
| *Pinus pinaster* | BX254814.1 | MASKIVLLLGICVFSFFLVSPVTVTEVGDAIGNNKTRSGPVEPPVSDHYVLGCKRCCYLDERGCISCCAKGDFAPTTAGRKEKAAIVAIDGHK |
| *Pinus sylvestris* | HE631983.1 | MASKIVLLLGICVFSFFLVSPVTVTEVGDAIGNNKTRSGPVEPPVSDHYVLNCKRCCYLDERGCISCCAKGDFAATTAGRNEKAAVVAIDGHK |
| *Pinus taeda* | DN630731.1 | MASKIVLVLGICVFAFFLLSPVTATEDGHAVEIKKEAQTMETPPGPVVPPRSEHYVSGCDRCCYLDERGCIACCSKGQ |
| *Pinus taeda* | CF399267.1 | MASKIVLVLGICVFAFLLLSPVTATEDDHTVEIKKEAQTMGTPPGPVVAPPRVVPPRSEHYVSGCNRCCYLDERGCIACCSKGQ |
| *Pinus taeda* | DR015859.1 | MASKIVLVLGICVFAFFLLSPVTATEDDHAVESKKEAQTMETPPGPVVPPRSEHYVSGCKRCCYLDERGCIACCSKGQ |
| *Pinus jeffreyi* | MFTM-2008250 | LVLGICVFAFFLLSPVTATEDDHAVESKKEAQTMETPTGPVVPPRSDHYVSGCKRCCYLDERGCIACCAKGDFAPTTAGQKEKAAVVANYGHK |
| *Pinus jeffreyi* | MFTM-2008251 | MASKTVLLLGICVFSFFLVSPVTVTEVGDAIENSKKLSGPVEPPVSDHYVIGCKRCCYLDERGCIACCAKGDFAPTTAGQKEKAAVVANYGHK |
| *Pinus parviflora* | IIOL-2011825 | MASKLVLVFGLCVFAILLLFPVTATEVDHAVIAAQTTKPADSDPAEPPVNDHYVTGCKRCCYLDERGCIACCPKGQL |
| *Pinus parviflora* | IIOL-2010776 | MVSKIVLVLGVCVFAFFMVFHVTALDDDHAIENNKTRSGPVLPPASDHYVSGCKRCCYLDERGCISCCSKGDFVSMTAGGKDKTAVVDNFGHK |
| *Pinus radiata* | DZQM-2007469 | MASKIVLVLGICVFAFLLLSPVTATEDDHAVEIKKEAQTMETPPGPVVPPRSLHYVSGCKRCCYLDERGCIACCSKGQ |
| *Platycladus orientalis* | BUWV-2043667 | MASKAVMAFTLCVFAVFLLQSSTSATAIDALFGESKAQEGPVVPDLQKHYVRDCKRCCYLDEYGCLSCCGKGQQL |
| *Podocarpus coriaceus* | SCEB-2046876 | MAVKAALVLALCLSALFLISSPVSAQVPSTTADPVVPPRADHYVTGCKRCCYLDERGCIRCCGKGELL |
| *Podocarpus rubens* | XLGK-2051806 | MAVKAALVLALCLSALFLISSPVSAQLPSTTPPEPVVPPRADHYVSGCKRCCYLDERGCITCCGKGQ |
| *Prumnopitys andina* | EGLZ-2034426 | MALKPALVLALCLSALLLVSPPVSAQPPSGGPPGAVIPDPADHYVTGCKRCCYLDERGCIACCGKGHLL |
| *Pseudolarix amabilis* | AQFM-2075847 | MASKVVLVLGICVFAFFLLSPVTAIEDDHAIEFKKEAQTAETPPEPVLPPRSEHYVRGCKRCCYLDERGCIACCAKGQ |
| *Pseudotaxus chienii* | YLPM-2072656 | MAVKVTLLSCFCIFSLFILLCAVSAIDSTDGIVVGDTKATAPPGPVAPPRRDHYETGCARCCYLDEWGCITCCTQAAGRKEKAAALPISIHQ |
| *Pseudotaxus chienii* | YLPM-2003219 | MASKIAMVFAVCIFALCLLQSSPSATVTAQVAETPPQGPVAPPLQKHYVRDCKRCCYLDEWGCLSCCGKAGQI |
| *Saxegothaea conspicua* | QCGM-2068475 | MAVKSALVLALCLSALFLVSTPVSAQVPSSPLPAQVVPPRADRYVYDCKRCCYLDERGCITCCGKGQQL |
| *Sequoiadendron giganteum-Glaucum* | QFAE-2043866 | MASKVVMVFTVCVFAVFLLQSSTSATATDALLGENKAQEGPVAPDLQKHYVRDCKRCCYLDEYGCLSCCGKGQQL |
| *Sundacarpus amarus* | KLGF-2088561 | MAIKPALVLALCLSALFLVSSPVSAQVPSTTPPVPVLPDPADHYVSGCKRCCYLDERGCIACCGKGQ |
| *Taiwania cryptomerioides* | QSNJ-2045938 | MVFTLCVFAVLLLESSTSATATDALFGENKAQEGPVAPDLQKHYVRDCKRCCYLDEYGCLSCCGK |
| *Taiwania cryptomerioides* | QSNJ-2056978 | MVFTLCVFAVFLLQSATSATATDALLGENKAQEGPVVPDRQKHYVKDCKRCCYLDEYGCLSCCGKGQQL |
| *Taxodium distichum* | FHST-2006246 | MASKVVMVLTLCVFTVFLLQSSTSATATDALFGENKAREGPVVPDRQKHYVRDCERCCYLDEYGCLSCCGKGQQL |
| *Taxodium distichum* | FHST-2006247 | MASKAVMVFTLCVFAVFILQSSVSATATDALFGENKAQEGPVVPDREKHYVRDCNRCCYLDEYGCLSCCGKGQQL |
| *Taxus baccata* | WWSS-2006951 | MASKIAMVFVVCIFALCLLQSSPSVTVTAQVTETPPQGPVVPPREKHYVRDCKRCCYLDEWGCLSCCGKAGQI |
| *Tetraclinis sp.* | CGDN-2059312 | MASKAAMVFTLCVFAVFLLQSQTSATAIDALFGESKAQEGPAVPDLQKHYVRDCKRCCYLDEYGCLSCCGKGQQL |
| *Torreya nucifera* | HQOM-2015294 | MAPKIAMVFAVCIFTLCLLQSSAPATAQVTETPPEGPVVPPRAKHYVRDCNRCCYLDERGCISCCGKGGQII |
| *Tsuga heterophylla* | GAMH-2000466 | MASKVILVLGICFLALFMLSPVTAIEDDHAVEIKKEAQTTPPPYEPILPPRSEHYVVGCKRCCYLDERGCIACCSKGGLQDLLPMTAGRKEKAALVANSGHQ |
| *Tsuga heterophylla* | GAMH-2002390 | MASKVVLVLGICVFALFLLSPVTAKEDDHAVGIEKEAQTAETPSGPVVPPRSEHYVNGCKKCCYLDERGCIACCSKGQL |
| *Welwitschia mirabilis* | TOXE-2053338 | MKRVSGSATVVCVVTLLLLISTQHFPATVSAQSGPAVPDRGTHYVRGCKRCCYLDEYGCIRCCSKGERLFSATDVPT |
| *Ginkgo biloba* | SGTW-2035618 | MASKIALLLVVCFCALFLISSATAGELENNKEIIDSSKPKGKTYPPGPVVPPRRDHYETGCKRCCYLDEYGCIRCCKEGEAATTTTTMDEPAAMTDSGHQ |
